# Colonization of distant organs by tumor cells generating circulating homotypic clusters adaptive to fluid shear stress

**DOI:** 10.1038/s41598-021-85743-z

**Published:** 2021-03-17

**Authors:** Manabu Maeshiro, Satoru Shinriki, Rin Liu, Yutaka Nakachi, Yoshihiro Komohara, Yukio Fujiwara, Kazuaki Ohtsubo, Ryoji Yoshida, Kazuya Iwamoto, Hideki Nakayama, Hirotaka Matsui

**Affiliations:** 1 Department of Molecular Laboratory Medicine, Graduate School of Medical Sciences, Kumamoto University, 1-1-1 Honjo, Chuo-ku, Kumamoto, 860-8556 Japan; 2 Department of Oral and Maxillofacial Surgery, Graduate School of Medical Sciences, Kumamoto University, Kumamoto, 860-8556 Japan; 3 Department of Molecular Brain Science, Graduate School of Medical Sciences, Kumamoto University, Kumamoto, 860-8556 Japan; 4 Department of Cell Pathology, Graduate School of Medical Sciences, Kumamoto University, Kumamoto, 860-8556 Japan; 5 Department of Biomedical Laboratory Sciences, Graduate School of Medical Sciences, Kumamoto University, Kumamoto, 860-8556 Japan

**Keywords:** Cancer, Cell biology, Oncology

## Abstract

Once disseminated tumor cells (DTCs) arrive at a metastatic organ, they remain there, latent, and become seeds of metastasis. However, the clonal composition of DTCs in a latent state remains unclear. Here, we applied high-resolution DNA barcode tracking to a mouse model that recapitulated the metastatic dormancy of head and neck squamous cell carcinoma (HNSCC). We found that clones abundantly circulated peripheral blood dominated DTCs. Through analyses of multiple barcoded clonal lines, we identified specific subclonal population that preferentially generated homotypic circulating tumor cell (CTC) clusters and dominated DTCs. Despite no notable features under static conditions, this population significantly generated stable cell aggregates that were resistant to anoikis under fluid shear stress (FSS) conditions in an E-cadherin-dependent manner. Our data from various cancer cell lines indicated that the ability of aggregate-constituting cells to regulate cortical actin-myosin dynamics governed the aggregates’ stability in FSS. The CTC cluster-originating cells were characterized by the expression of a subset of E-cadherin binding factors enriched with actin cytoskeleton regulators. Furthermore, this expression signature was associated with locoregional and metastatic recurrence in HNSCC patients. These results reveal a biological selection of tumor cells capable of generating FSS-adaptive CTC clusters, which leads to distant colonization.

## Introduction

Metastasis, which is responsible for > 90% of cancer-related deaths, is caused by disseminated tumor cells (DTCs) that initiate new tumors at distant organ sites. However, the metastatic mechanism still remains unclear^1^. Mounting evidence suggests that after settling into distant organs, DTCs effectively remain dormant until a trigger, as yet unknown, rouses them^2^. Clinically, although most DTCs in bone marrow (BM) are negative for proliferation markers^3^, the abundance of these cells directly correlates with reduced metastasis-free survival in various types of cancer including head and neck squamous cell carcinoma (HNSCC)^3^. Despite such biological and clinical implications, the composition of DTCs surviving in a latent state remains obscure.

In fact, successful cancer cell colonization depends on the ability of circulating tumor cells (CTCs) to infiltrate and survive in the target tissue^4^. However, most CTCs have a short half-life in circulation and are apoptotic because of a combination of mechanical and environmental trauma including fluid shear stress (FSS), oxidative stress, attack by the immune system, lack of growth factors, and anoikis^5,6^. Recent studies have shown that CTCs are structurally and phenotypically heterogeneous^7^. Clusters of CTCs have been detected in the peripheral blood (PB) of patients with various types of cancer including HNSCC^8,9^ and have been demonstrated to be highly metastatic compared with individual CTCs^10–12^. Elucidating how heterogeneous CTCs contribute to distant colonization and clonal diversity within DTCs is important for better understanding of mechanisms underlying tumor progression and targeting metastasis.

Recent studies of tumor heterogeneity by using cell tracking and single-cell sequencing have begun to demonstrate clonal divergence between primary tumors and metastases^13–15^. Of various tracking techniques, the DNA barcode method has a general advantage with regard to sensitivity^16^, thereby having the potential to illustrate clonal compositions of rare populations such as CTCs and DTCs, even when latent. However, of particular concern is that most studies using such tracking methods and genomic sequencing were retrospective. Hence, genetic or visual variation could not directly point to specific biological contributions of clonal populations to disease progression. In the current study, we therefore applied prospective analyses of subclones to a DNA barcode tracking strategy in a latent DTC mouse model. We identified a stable functionally homotypic CTC clustering by specific tumor cells that regulated actomyosin machinery in response to FSS, as a crucial clonal selection process leading to dominant colonization of distant organs.

## Methods

### Cells and 2D cell culture

The human HNSCC cell line SAS was donated by the Cell Resource Center for Biomedical Research, Tohoku University (Sendai, Japan). MCF-7 and MDA-MB231 were purchased from ATCC; Ca9-22 and KN from Japanese Collection of Research Bioresources (JCRB, Osaka, Japan). HOC-313 and TSU were kind gifts from Drs. Shuichi Kawashiri and Koroku Kato (Kanazawa University, Ishikawa, Japan). SAS cells expressing copGFP and firefly luciferase (SAS-GL) or mCherry were generated via lentiviral transfection of CMV-GFP-T2A-Luciferase (BLIV101PA-1; System Biosciences, Mountain View, CA) or pmCherry (632522; Clontech, Rockville, MD), respectively. SAS-GL and the other cell lines were grown in RPMI1640 (Sigma Aldrich, St. Louis, MO) and DMEM (Thermo Fisher Scientific, Waltham, MA) supplemented with 10% heat-inactivated FBS (Thermo Fisher Scientific) in 5% CO_2_ at 37 °C, respectively. Unless stated otherwise, cells were cultured on adherent tissue culture plates (Greiner Bio-One, Frickenhausen, Germany). SAS and Ca9-22 were authenticated by short tandem repeat profiling. All cells were examined for Mycoplasma routinely by PCR.

### Animal studies

All the animal experiments conformed to the Animal Research: Reporting of In Vivo Experiments (ARRIVE) guidelines^17^ and were approved by The Animal Care and Use Committee (ACUC) of Kumamoto University (Certificate No. A2019-055). All the experiments were performed under an approved ethical procedure and guidelines provided by ACUC of Kumamoto University. Crlj:SHO-Prkdc^scid^ Hr^hr^ (SHO) mice at 6–8 weeks old were purchased from Charles River Japan (Yokohama, Japan) and were maintained at the Center for Animal Resources and Development of Kumamoto University according to the approved ACUC protocol. Each mouse received orthotopic injections of 1 × 10^6^ human tumor cells into the buccal mucosa. For limited 32-clone transplantation, each clone was adjusted to the same number, and 1 × 10^6^ cells of the mixed clones were injected. Bioluminescent imaging was performed 15 min after intraperitoneal injection of 3 mg D-luciferin (eBioscience, San Diego, CA) by using Night Owl II LB983 (Berthold Technologies GmbH, Bad Wildbad, Germany).

The PB samples of the mice were collected by means of retro-orbital sinus puncture at the corner of the eye socket, with clean 75-mm heparinized microhematocrit tubes used (Drummond Scientific Company, Broomall, PA), before they were killed 30 days after the injection. Primary tumors and femur were extracted according to the methods used for rare DTCs detection^18,19^ with small modifications. In details, primary tumors were minced with a surgical scalpel and homogenized by using FastPrep FP120 (Q-BIOgene, Irvine, CA) with MP Biomedicals Lysing Matrix D (Thermo Fisher Scientific) in 200 μL of Hanks' balanced salt solution. Femurs were separated from the body at the joints. After skin and muscle were removed with scissors, both ends of the bone were excised, and BM was extracted by centrifugation at 1000×*g* in a double-structured 1.5-mL tube for 15 s (The double-structured tube is a nested 0.5-mL tube with an 18-G needle hole at the tip). For transplantation of the mixture of GFP- and mCherry-expressing cells, two distinct populations (5 × 10^5^ cells/population) were mixed and then a total of 1 × 10^6^ cells was orthotopically injected into the buccal mucosa of SHO mice. After 30 days, PB, primary tumor, and BM samples were collected as described above.

### Barcode library preparation and lentiviral transduction

The ClonTracer library was a gift from Dr. Frank Stegmeier (Addgene #67267). Construction of the library was previously described^16^. The lentiviral barcode library was packaged by using HEK293T cells. Cells were plated on 10-cm adherent tissue culture plates (Corning, Corning, NY) to 70% confluency. A transfection mixture was prepared with barcode plasmid vector, psPAX2, and pMD2.G in Opti-MEM (Thermo Fisher Scientific). Transfection was performed by using TransIT-293 Reagent (Mirus Bio LLC., Madison, WI). Pools of 1 × 10^7^ SAS-GL cells were barcoded by lentiviral infection at a multiplicity of infection of 0.1, and infected cells were selected with puromycin (1.5 μg/mL). Infected cell populations were expanded in culture for the minimal time to obtain a sufficient number of cells for the animal experiments.

### Barcode analyses

Genomic DNA was isolated via NucleoSpin Tissue (Takara Bio, Otsu, Japan) for all tissues except blood. Genomic DNA of blood was isolated by using the QIAamp DNA Blood Mini Kit (QIAGEN, Hilden, Germany). Genomic DNA extracted from tumor cells contained a 30-bp semirandom barcode array that allowed multiplexing with standard Illumina MiSeq (Illumina, San Diego, CA) chemistry and software. After library preparation (see supplementary information), a dual-indexed single-read sequencing run (1 × 100 bp) was performed to generate Illumina FASTQ files. We carried out barcode-composition analysis as previously described^16^ (https://www.addgene.org/pooled-library/clontracer/). Briefly, sequencing reads were trimmed and then filtered to include only 30-nt reads that match the expected WS × 15 patterns. The barcodes with only one count were excluded from the analyses to avoid the noise derived from the sequencing error.

### Evaluation of BM-DTCs and CTCs in mice

PB samples from each mouse were processed for hemolysis by using BD Pharm Lyse (BD Biosciences, San Jose, CA). After centrifugation, BM and PB cells were fixed with 1% paraformaldehyde for 4 min at room temperature. The fixed cells were attached to Matsunami Adhesive Silane-coated glass slides (Matsunami Glass, Osaka, Japan) by using Cytospin (Thermo Fisher Scientific) and were briefly air-dried. Cell nuclei were stained with DAPI (Sigma Aldrich). ProLong Diamond Antifade Mountant (Thermo Fisher Scientific) was used to coverslip the slides. For each mouse, GFP- or mCherry-positive single cells, clusters, and cells within each cluster in 50 μL of PB and BM for half of the femur were counted. Totals of the number of cells including those in clusters and the clusters themselves in each mouse were obtained, and then average numbers and color compositions were calculated. Proportions of tumor cells positive for GFP or mCherry in primary tumors were calculated by using ImageJ software. Myosin IIA distribution in CTC clusters (n = 3 mice, ≥ 5 clusters/mouse) were evaluated by immunofluorescence (see supplementary information).

### Suspension cell culture in FSS

Single-cell suspensions at a density of 5 × 10^3^/mL unless otherwise indicated were plated on poly (2-hydroxyethyl methacrylate) (poly-HEMA)-coated plates and were then cultured under static conditions at 37 °C for 18 h. To evaluate FSS effects on cells, single-cell suspensions at a density of 5 × 10^3^/mL in DMEM/F12 were cultured on top of an orbital shaker (Model BR-42FL; Titec Co., Saitama, Japan) at ~ 2 dyn/cm^2^ (68 rpm) at 37 °C as previously described with modifications^20–22^. Cells were centrifuged and plated in 96-well plates, and cell aggregates (≥ 2 cells) were counted. Alternatively, cells were stained with an Annexin V/Alexa Fluor 647 conjugate (Thermo Fisher Scientific) or DAPI (Sigma Aldrich), centrifuged, plated in 96-well plates, and observed with Biozero BZ-8100 microscope (Keyence, Osaka, Japan). Annexin V- or DAPI-positive single cells and aggregates were counted. When indicated, adherent cells were pretreated with Latrunculin A (LatA, 1 μM), Blebbistatin (Bleb, 20 μM), NSC23766 (10–100 μM), or Y-27632 (10–100 μM) for 3 h, followed by culture in suspension with rotation as described above. Effects of short-term treatment with these inhibitors on cell viability were assessed by Annexin V staining or measuring Caspase 3/7 activity (see Supplementary information).

We evaluated the clonality of cell aggregates generated after suspension of the mixture of different colored populations. GFP-expressing cells (Parental or clone W cells) were labeled by using CellTrace Far Red Cell Proliferation Kit (Thermo Fisher Scientific) according to the manufacturer’s protocol. Far-red-labeled cells or Parental-mCherry cells were mixed with GFP-expressing cells, each at a density of 2.5 × 10^3^/mL, before starting suspension culture.

### Non-invasive capture of cell aggregates

CELLNETTA was provided from Murata Manufacturing Co. Ltd. (Kyoto, Japan). This apparatus is a metal mesh element which has sub-micrometer square apertures arrayed in a periodic manner. Cell aggregates were non-invasively captured by CELLNETTA according to the manufacturer’s instructions. Briefly, immediately after suspension culture under FSS conditions, the medium containing cells were passed through the mesh apparatus put on the 50-mL conical tube on the condition so that cell number per passing did not exceed approximately 5 × 10^5^. The mesh apparatus was then inverted over another conical tube, followed by fresh medium being passed from the back side of the mesh to recover the captured aggregates.

We have independently evaluated the performance of different CELLNETTA (10-, 15-, and 20-μm mesh size) using SAS-GL parental cells. We have confirmed that almost only aggregates were captured on the 20-μm mesh (see Supplementary Fig. S7l) even though some doublets passed through it. When using 15-μm mesh, relatively large single cells were also captured although small aggregates such as doublets were more efficiently captured than using 20-μm mesh. Accordingly, to recover only aggregate-derived cells, we used the 20-μm mesh in this study.

### Statistical analysis

Statistical significance was defined as *p* < 0.05 for Student’s *t* test and χ^2^ test. Student’s t test was used to compare means of two groups. JMP software Version 13 for Windows was used for statistical analysis.

## Results

### A limited number of clones dominates CTCs and DTCs

**Figure 1:**
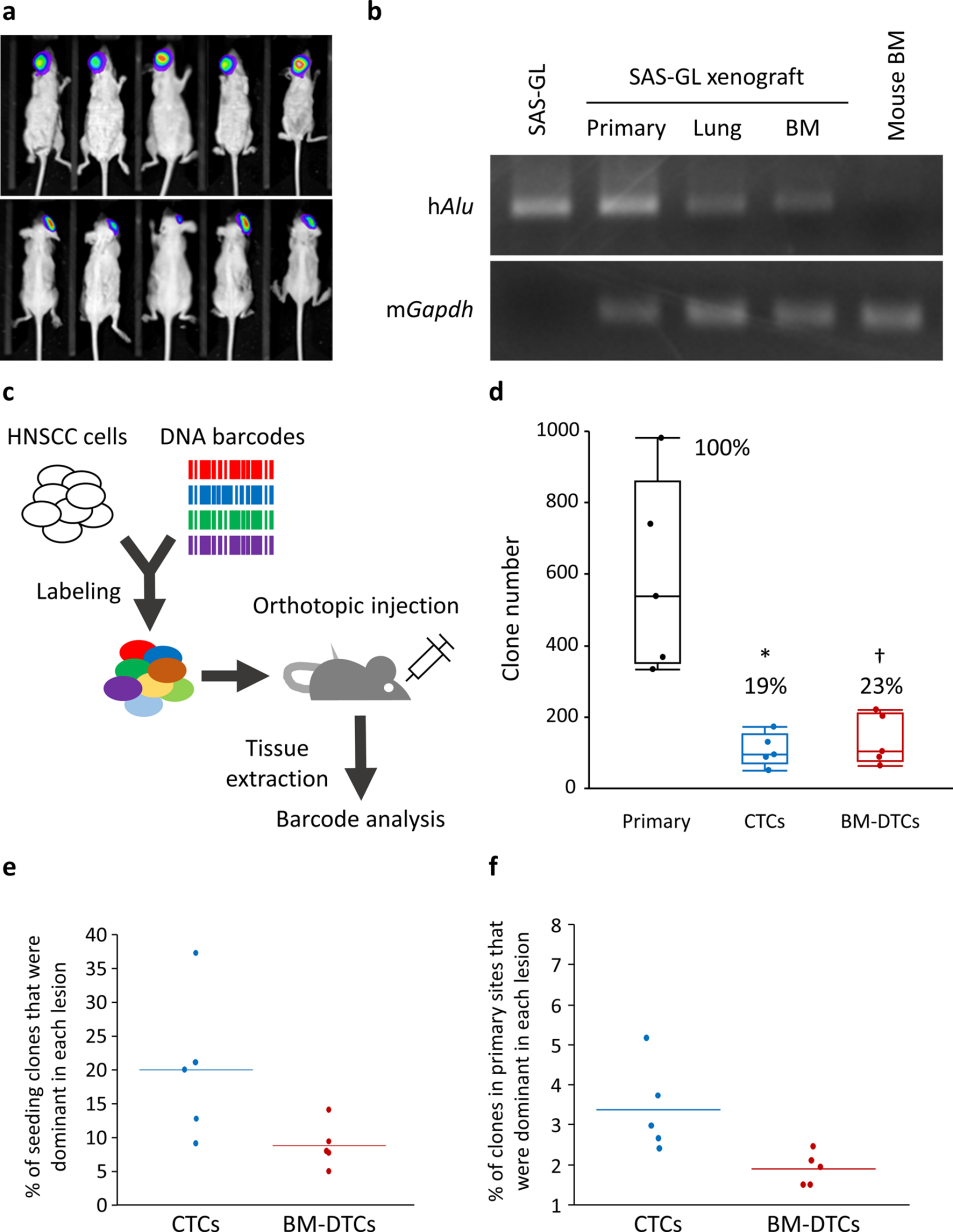
A limited number of clones dominates CTCs and DTCs. (**a**) Representative ventral (upper panels) and dorsal (lower panels) bioluminescent images of mice 30 days after orthotopic injection of SAS-GL cells. (**b**) Representative data for human *Alu* (h*Alu*) sequence amplification 30 days after orthotopic injection of SAS-GL cells that indicated SAS-derived DTC presence. Cultured SAS-GL cells (left end) and BM from nontransplanted mice (right end) were controls. m*Gapdh*, mouse *Gapdh*. Uncropped gels can be found in Supplementary Information. (**c**) Lentiviral barcode strategy for identifying clonal populations in SAS cells. (**d**) Unique seeding clones in the primary tumor, CTCs, and BM-DTCs were quantified and plotted. Above the bars are the percentages of unique clones in each organ site and the primary tumor. Values are means ± SEM (*n* = 5 mice), *p < 0.005; ^†^*p* < 0.01 (two-tailed t tests). (**e**) Percentages of dominant clones (top 75%) relative to the total number of seeding clones in each lesion. (**f**) Percentages of dominant clones (top 75%) in each lesion relative to the total number of unique clones in the primary tumor.

First, cells of the human HNSCC cell line SAS-GL were orthotopically injected into the buccal mucosa of SHO mice. Thirty days after the injection, we confirmed primary tumors as clear masses by visual inspection and bioluminescent imaging; we found no metastatic mass in other organs (Fig. 1a). However, even at this time point, our analysis via human *Alu* sequence-specific PCR revealed that DTCs occurred in the BM and lungs (Fig. 1b). All the BM-DTCs we visually detected were negative for a cell proliferation marker Ki-67 (Supplementary Fig. S1a), suggesting that BM-DTCs were in a dormant cellular state. In addition, no metastatic mass was found at least until the end of the observation period. We therefore believe that this xenograft model was a useful experimental system for analyzing the clonal architecture of latent DTCs.

To verify clonal distribution in the systemic circulation, we marked individual SAS-GL cells with a DNA barcode library via lentiviral infection. We then introduced a mixture of barcode-labeled SAS-GL cells orthotopically into five SHO mice. We estimated that approximately 740 barcodes in total with at least more than 100 cells per barcode were injected. Thirty days after the implantation, we extracted primary tumors, PB, and BM, and we quantified individually barcoded tumor cell clones within each tissue by massive parallel sequencing (Fig. 1c). We successfully detected tumor cell-derived barcodes in samples from all the organs with a sufficient number of reads (primary tumor, 0.52 ± 0.12 M; PB, 0.62 ± 0.21 M; BM, 0.53 ± 0.34 M). We found an average of 592 unique barcoded clones (80% of the total injected clones) in primary tumors (Fig. 1d). The number of clones detected in the PB (CTCs) and BM (BM-DTCs) was significantly low, with an average of 107 (19% of all clones in the primary site) and 136 (23%), respectively (Fig. 1d). Only 19 clones (20% in CTCs) and 11 clones (9% in BM-DTCs) of these seeding clones accounted for the top 75% of clones of each organ (defined as dominant clones; Fig. 1e) with the sequencing coverage per sample ranging from 2.3 to 221 K reads (Supplementary Fig. S1b). Furthermore, these dominant clones were derived from only 3.4% (CTCs) and 1.9% (BM-DTCs) of the clones in the primary tumors (Fig. 1f). These results indicated that only specific clones in the primary tumors were able to dominate CTCs and DTCs.

### Abundance within CTCs correlates with dominant distant colonization

**Figure 2:**
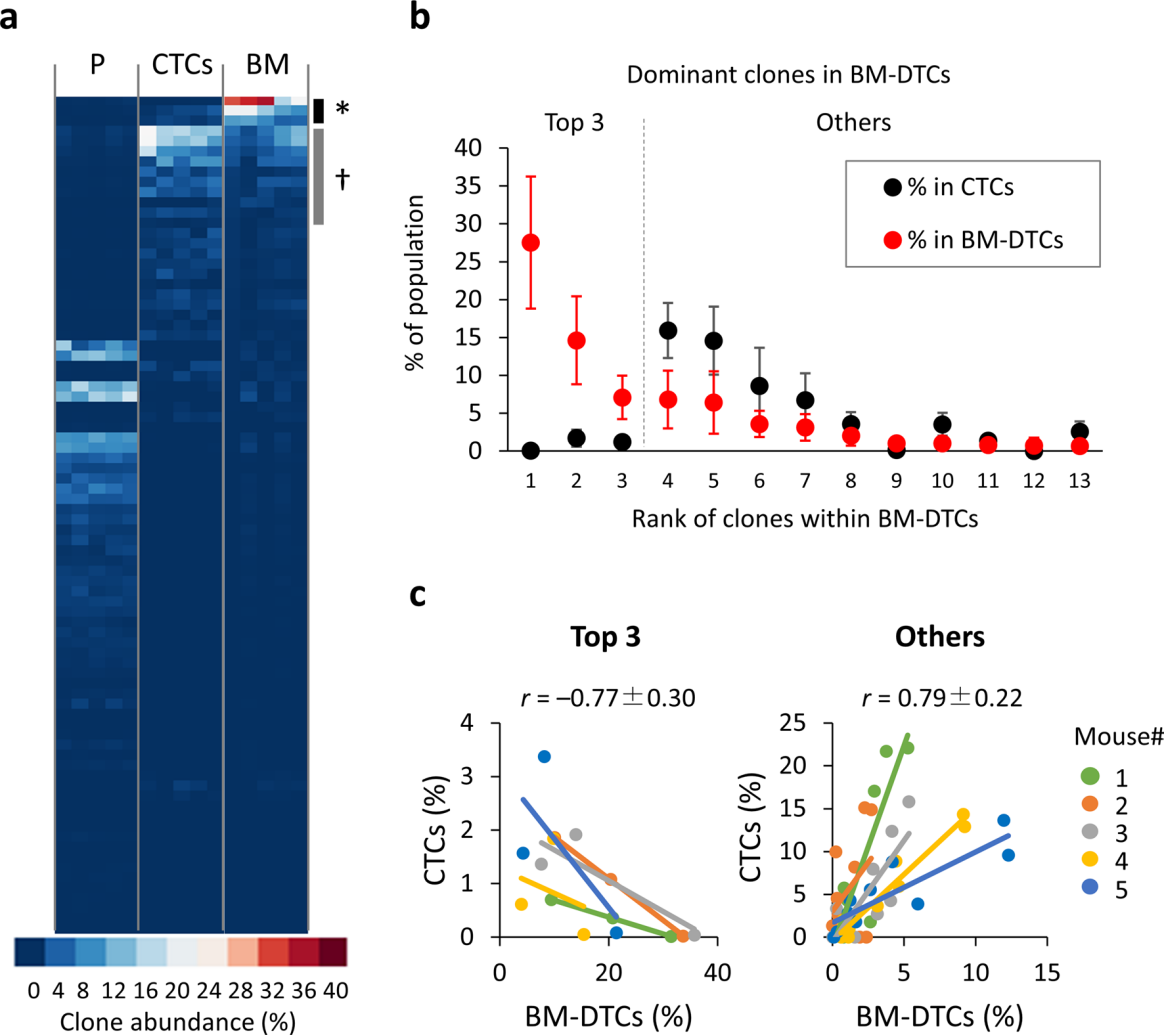
DTC clonal architecture associated with abundance of CTCs. (**a**) Relative proportions of clones that engrafted in mice (*n* = 5) at the primary tumors (P), CTCs, and BM. The asterisk and dagger indicate the top 3 abundant clones and other dominant clones in BM-DTCs, respectively. (**b**) Abundance of dominant clones (top 75%, on average) in BM-DTCs (red) and that of corresponding clones in CTCs (black). (**c**) Correlation of abundance of dominant clones (left panel: top 3 clones; right panel: other dominant clones) in BM-DTCs to that in CTCs. Each dot represents one sample from an individual mouse.

We next asked whether the subset of clones that engrafted at all primary sites across all five mice (82 clones) showed consistent behavior in terms of disease progression. All other clones were either undetected or negligible in most mice (Supplementary Fig. S2a). The selected 82 clones showed strong correlation for abundance among mice in each organ (Supplementary Fig. S2b). Rare clones rather than prominent clones within primary tumors dominantly contributed to clonal architecture of CTCs and DTCs (Fig. 2a). Although the clonal profile of BM-DTCs showed a modest correlation with that of CTCs (Supplementary Fig. S2c), the dominant clones (top 75%, on average: *n* = 13) in BM-DTCs were divided into two groups (Fig. 2a,b). The first group consisted of the top three dominant clones occupying half (49%) of the DTCs but rare even in CTCs (0.04–1.71%) (Fig. 2b,c, left panel; Supplementary Fig. S2d), suggesting that these clones have outstanding potentials specialized to adapt BM microenvironment. The second group comprised the remaining dominant clones whose abundance correlated well with those in CTCs (Fig. 2b,c, right panel). The highly reproducible behavior of each clone indicated that clonal dominance observed in CTCs and DTCs was attributed to the distinct intrinsic properties of each clone.

### Identification of an origin clone dominating CTCs and DTCs

**Figure 3:**
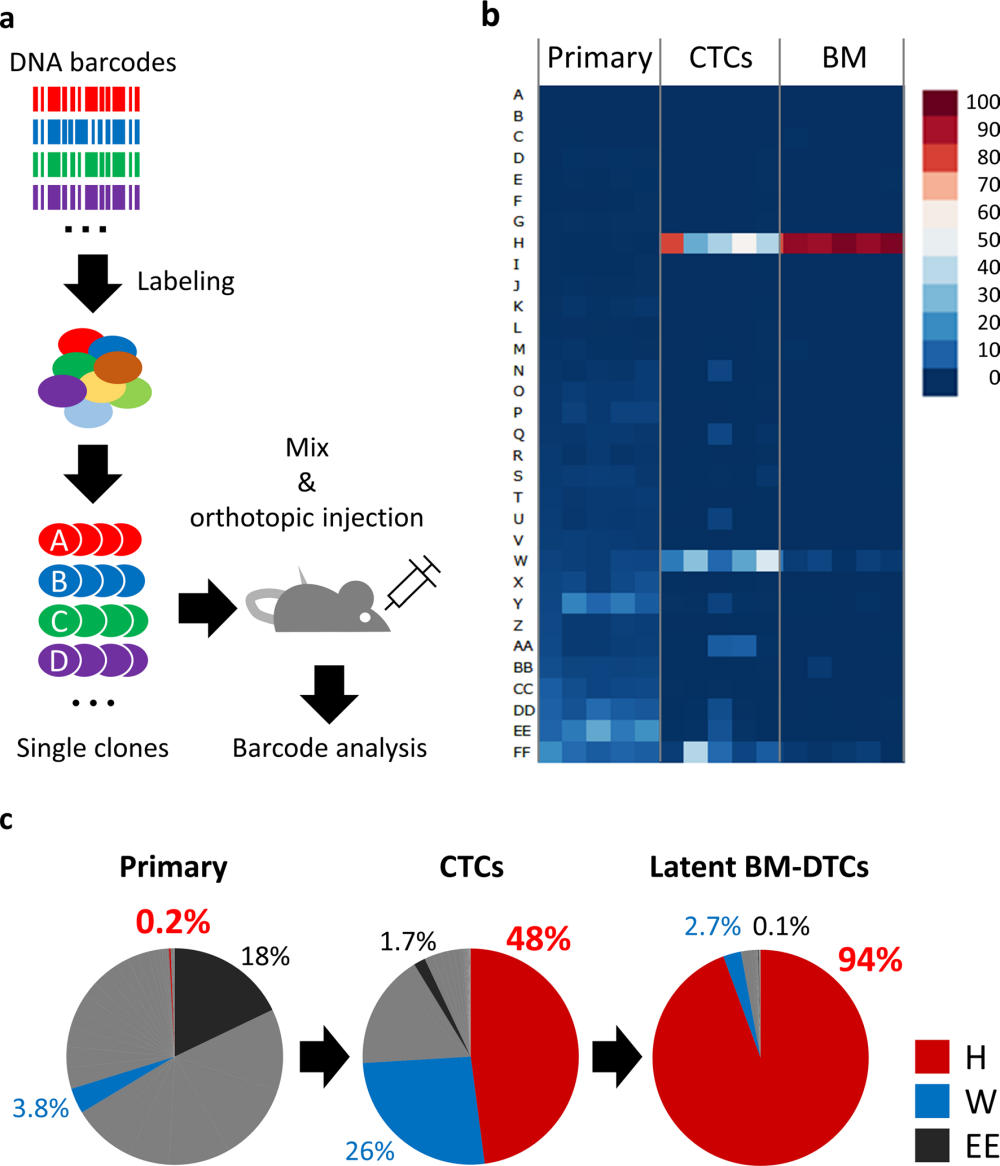
Identification of a clone dominating CTCs and DTCs. (**a**) Strategy for isolating individual SAS-GL clones with molecular barcodes. (**b**) Relative proportions of clonal lines among primary tumors, CTCs, and BM in mice (*n* = 5). (**c**) Pie charts showing abundance of clone H (red), clone W (blue), and clone EE (black) in primary tumors, CTCs, and latent BM-DTCs in mice. The mean percentage of each clone is given for each chart.

We therefore wished to understand the properties of clones with high competence for CTCs and DTCs. To this end, we established 32 single-cell-derived clonal lines from another barcoded SAS-GL population (Fig. 3a; Supplementary Fig. S3a). After minimal propagation, the clones were pooled and orthotopically injected into SHO mice (*n* = 5). Clonal profiling for each organ by barcode analysis revealed the presence of two dominant clones, “H” (48%, on average) and “W” (26%, on average), in CTCs (Fig. 3b,c; Supplementary Fig. S3b). Of these, clone H occupied most of the populations in BM-DTCs (94%). This behavior of clone H appeared to represent that of clones already observed when the parental population (SAS-GL) was injected (Fig. 2). Although clone W was the second most abundant clone in BM, its seeding capacity was evidently much lower than that of clone H. Clone EE, the most abundant clone in the primary lesion (18%), showed a quite low capacity to seed CTCs and DTCs (Fig. 3b,c).

We first screened the phenotypic characteristics of these specific clones by using a 2D culture system. In this system, clones H, W, and EE had a round clustered epithelial organization shape similar to the parental population (Supplementary Fig. S3c). In addition, no obvious increase in cell proliferation and solitary invasion occurred in clone H (Supplementary Fig. S3d and Supplementary Fig. S3e). Therefore, given the rare occurrence (only 0.2%) of clone H in primary tumors (Fig. 3b,c), the significant enrichment of clone H in CTCs likely resulted from properties that were distinct from cell growth and solitary invasion in the primary site.

### A DTC-originating clone efficiently forms anoikis-resistant aggregates under FSS conditions

**Figure 4:**
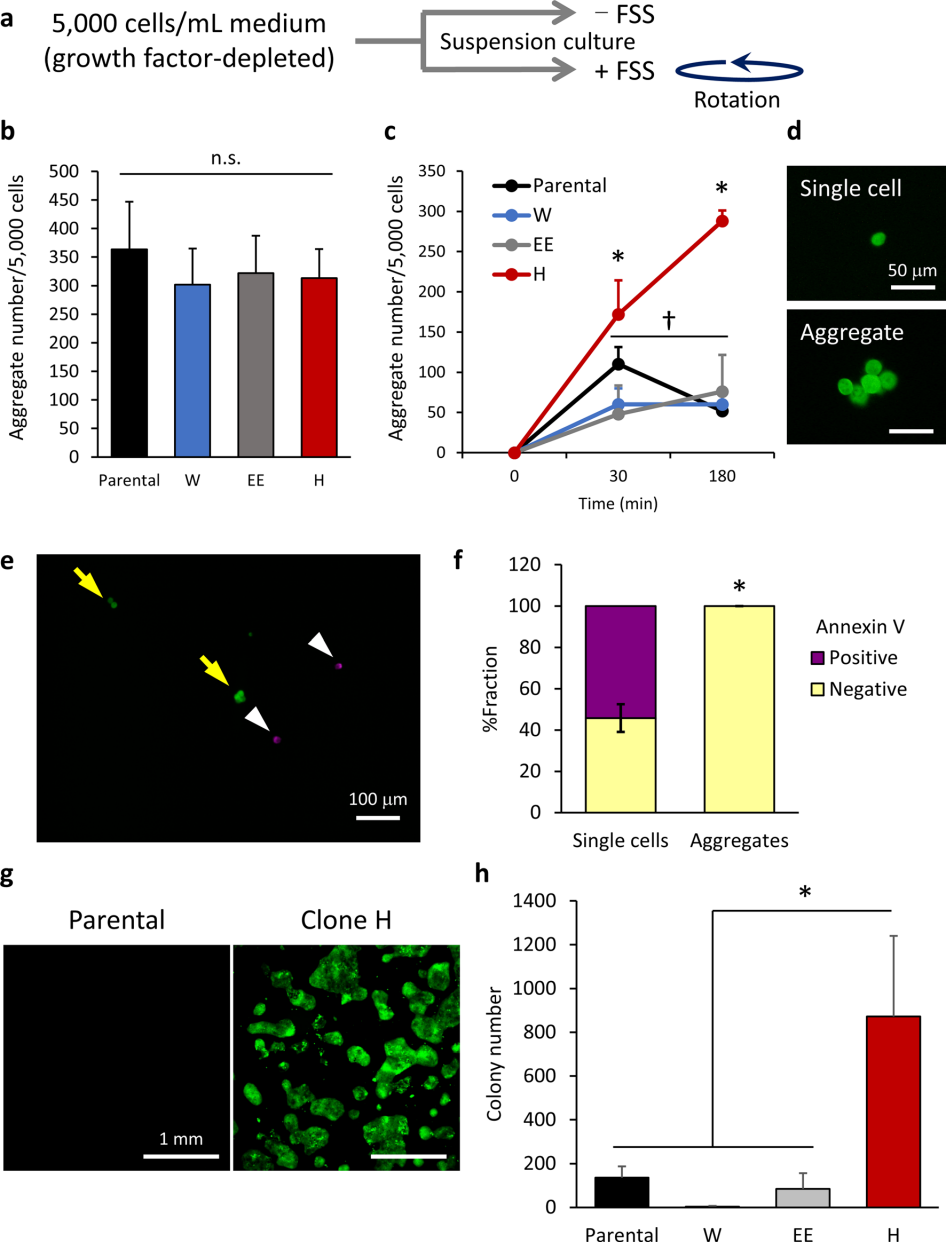
A DTC-dominating clone efficiently forms anoikis-resistant aggregates under FSS conditions. (**a**) Diagram showing the experimental setup of the anoikis assay. Single cells (5 × 10^3^) obtained from SAS-GL cells (parental), clone W, and clone H were separately suspended in culture medium and then cultured on poly-HEMA-coated plates under static conditions (−FSS) or rotated (+FSS) at 37 °C. (**b**) Numbers of aggregates in suspension culture at 18-h under static conditions. Values are means ± SEM of triplicate samples. n.s., not significant. (**c**) Numbers of aggregates generated in suspension culture under FSS conditions. Values are means ± SEM of triplicate samples. *p < 0.005 (two-tailed t tests). (**d**) Representative images of single cells (upper panel) and aggregates (lower panel) derived from clone H cultured under FSS conditions. (**e**) Annexin V staining with clone H cells at 3 h of suspension culture. Arrows and arrowheads indicate annexin V-negative aggregates and -positive single cells, respectively. (**f**) Proportion of annexin V-positive and -negative cells in single cells and aggregates. *p < 0.0001 (Pearson's χ^2^ test). (**g**,**h**) Single cells (5 × 10^4^) were suspended in culture medium and rotated for 22 h at 37 °C. Cells were centrifuged, plated in 6-well plates, and cultured for 6 days. Colonies of surviving cells with areas > 2500 μm^2^ were counted. (**g**) Representative images of colonies. (**h**) Number of colonies derived from parental cells and clones W, EE, and H. Values are means ± SEM of triplicate samples. *p < 0.05 (two-tailed t tests).

A majority of CTCs are assumed to exhibit anoikis^6^. Intravascular death of tumor cells can also result from mechanical forces related to FSS and loss of growth factor in the bloodstream^6,23^. To investigate the involvement of these factors in the behavior of clone H in circulation, we used a suspension cell culture, with cells grown in growth factor-depleted DMEM/F12 medium under static conditions or under FSS conditions^20–22^ (Fig. 4a). Without FSS, the parental cells and clones W, EE and H equally formed cell aggregates at various cell concentrations (Fig. 4b; Supplementary Fig. S4a). However, under FSS conditions, only clone H efficiently formed cell aggregates in 30 min, and the number of aggregates kept increasing for 3 h (Fig. 4c,d; Supplementary Fig. S4b). Although the parental population, clone W, and clone EE also formed lower numbers of aggregates in 30 min under FSS conditions, thereafter the aggregate number was significantly reduced or did not change.

As a striking finding, although we observed many apoptotic cells (anoikis) in clone H at 3 h in the suspension culture with FSS, which mimicked a short half-life of less than 2.5 h of CTCs in circulation^5^, annexin V-positive apoptotic cells (Fig. 4e,f) and DAPI-positive dead cells (Supplementary Fig. S4c and Supplementary Fig. S4d) were always single cells, and 100% of the cells in aggregates were negative for both markers independently of cluster size. Similar results were observed in the heterogeneous parental population, clone W, and clone EE (Supplementary Fig. S4e), although the number of aggregates in these populations was much smaller than that of clone H (Fig. 4c). There was no survival advantage for cell aggregates generated in suspension cultures under static conditions (Supplementary Fig. S4f).

We then analyzed the colony-forming capacity after an anoikis challenge in FSS, and found that clone H formed colonies more than 6, 10 and 230 times more efficiently than the parental population, clone EE and clone W, respectively (Fig. 4g,h). Together our data indicated that clone H has an intrinsically preeminent capacity to form and maintain anoikis-resistant cell aggregates under the floating conditions in FSS, which allows their efficient colonization after exposure to such a harsh environment.

### Clonal selection by homotypic cell aggregation

**Figure 5:**
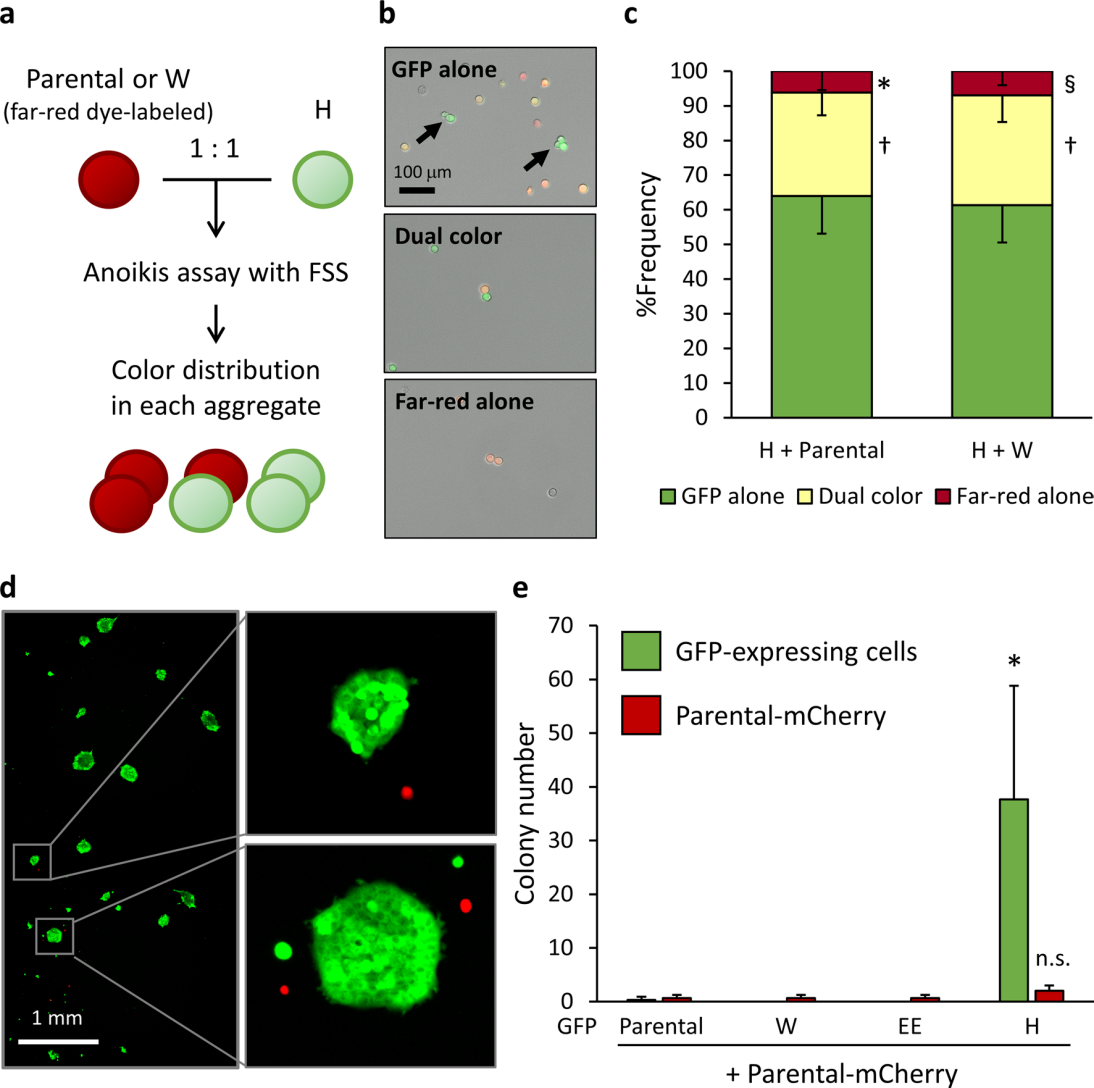
Clonal selection by homotypic cell aggregation in suspension with FSS. (**a**) Equal numbers of far-red-labeled GFP-expressing parental cells or clones W and H cells were mixed and suspended in culture medium followed by rotation for 3 h at 37 °C. (**b**) Representative images of cell aggregates composed of only GFP-expressing cells (upper), the mixture of GFP-expressing cells and far-red-labeled cells (middle), and only far-red-labeled cells (bottom). (**c**) Abundance ratio of each aggregate type. Error bars represent SEM of triplicate samples. *p < 0.005; ^†^*p* < 0.01; ^§^*p* < 0.0001 vs. GFP alone in each experimental group (two-tailed t tests). (**d**,**e**) Equal numbers of mCherry-expressing parental (Parental-mCherry) cells and GFP-expressing parental cells and clones W, EE, or H were mixed and suspended in culture medium followed by rotation for 22 h at 37 °C. Cells were centrifuged, plated in 6-well plates, and cultured for 6 days. Colonies of surviving cells with areas > 2500 μm^2^ were counted. (**d**) Representative images of colonies derived from the parental-mCherry and clone H cell mixture. (**e**) The number of GFP- or mCherry-positive colonies in each experimental group. Values are means ± SEM of triplicate samples. *p < 0.05 (vs. all other GFP-positive and mCherry-positive cells); n.s., vs. mCherry-positive cells in all other groups.

To determine whether clone H involves or engages other tumor cell populations with different intrinsic properties to promote their circulation or seeding at secondary sites, we suspended a mixture of clone H cells expressing GFP and far-red dye-labeled parental cells (SAS-GL) or clone W cells under FSS conditions (Fig. 5a,b). Strikingly, the cell aggregates that were generated consisted primarily of only clone H, which reflects a preferential homotypic cell aggregation (Fig. 5c). Thus, after the anoikis challenge, even with mCherry-expressing parental cells, only clone H formed colonies on adherent culture plates (Fig. 5d,e; Supplementary Fig. S5). Clones W and EE showed extremely low colonization efficiency, at the same level as the mixed mCherry-expressing parental cells (Fig. 5e; Supplementary Fig. S5). These results agreed with the barcode composition results for CTCs and DTCs (Fig. 3b). These data indicated clonal selection via homotypic cell aggregation during suspension culture under FSS conditions.

### Dominant distant colonization via homotypic CTC clusters

**Figure 6:**
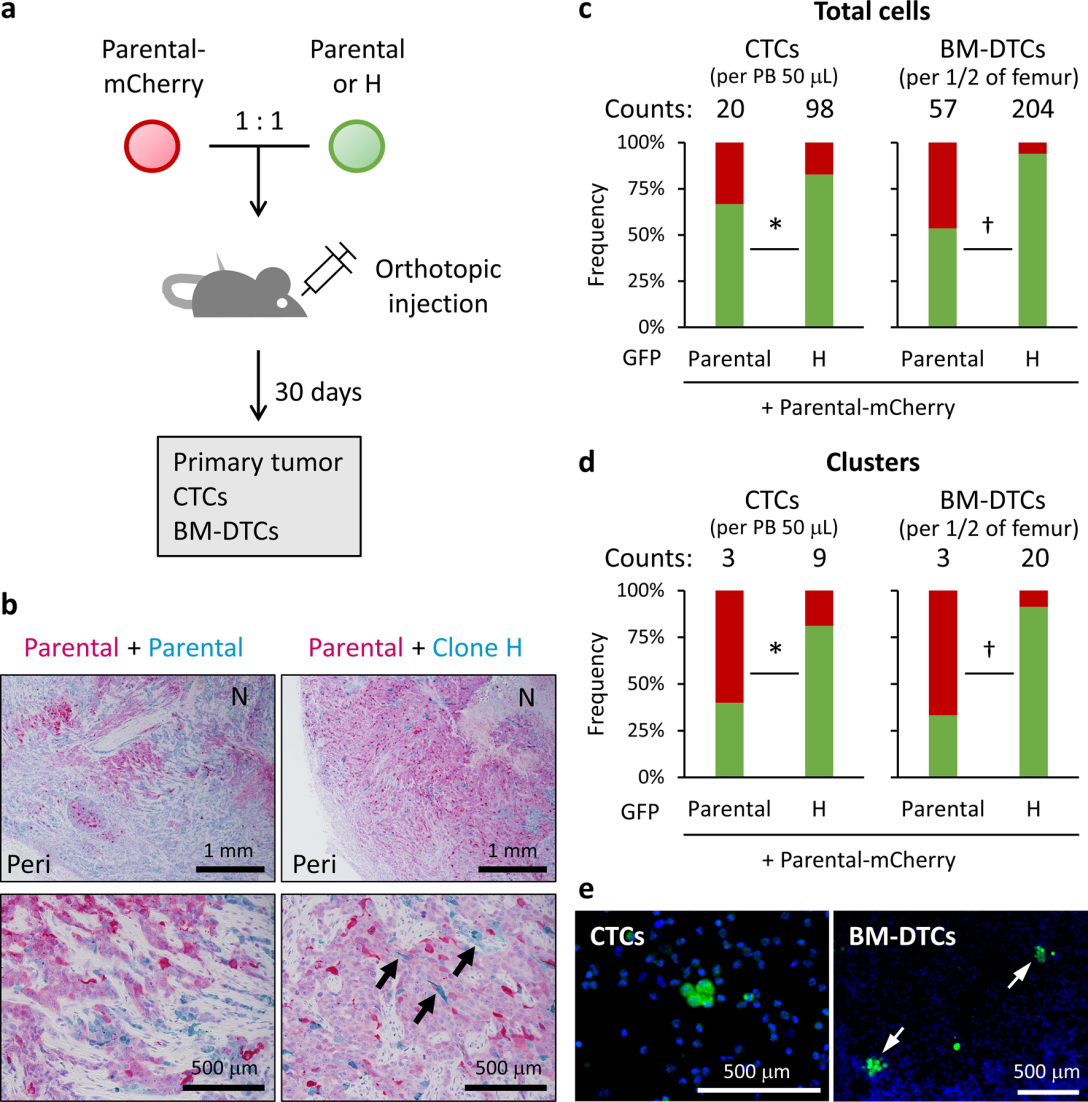
Dominant distant colonization by homotypic CTC clusters. (**a**) Equal numbers of Parental-mCherry and GFP-expressing parental cells (Parental) or clone H were mixed and orthotopically injected into mice. After 30 days, numbers of GFP-positive or mCherry-positive cells or clusters in PB (CTCs) and BM-DTCs were counted. (**b**) Representative images of primary tumors from mixtures of parental/parental cells (left panels) and parental/clone H cells (right panels). Arrows indicate cells originating from clone H. Peri: peripheral lesion. N: necrotic lesion. (**c**,**d**) Proportions of total cells (**c**) and cell clusters (**d**) positive for only GFP (green) or mCherry (red) in CTCs (left panels) and BM-DTCs (right panels) were calculated for each pooled sample. Average numbers per 50 μL of PB (right panels) and BM for half of the femur (left panels) from each mouse are given above each bar. *p < 0.05; ^†^*p* < 0.0005 (Pearson's χ^2^ test). (**e**) Representative images of clusters in CTCs (left panel) and BM-DCTs (right panel).

To validate our in vitro findings, we orthotopically injected mice with mCherry-expressing parental cells after mixing the cells with an equal number of GFP-expressing clone H or parental cells (Fig. 6a). In the primary tumors, cells comprising clone H (GFP-positive area) appeared to be relatively few compared to the mixed parental population (mCherry-positive area) (Fig. 6b, upper panels; Supplementary Fig. S6a). We did not find convincing evidence of preferential cell invasion for clone H in the primary tumors (Fig. 6b, lower panels).

As expected, the numbers of visibly detectable CTCs and BM-DTCs that originated from the mixture of the two parental populations were small (Fig. 6c). In the mixture containing clone H, however, the numbers showed a fivefold or fourfold increase, on average, per sample in CTCs and BM-DTCs, respectively. Despite the injected mixture being highly heterogeneous and a few occurrences of clone H in the primary site, both CTCs and BM-DTCs were primarily occupied by clone H, similar to our data from clonal tracking and in vitro experiments (Figs. 3, 4 and 6c). Furthermore, the numbers of clusters in CTCs and BM-DTCs were also threefold and sevenfold higher in the animals injected with clone H, respectively (Fig. 6d). In support of our in vitro findings, clone H alone predominated in most clusters (Fig. 6d,e). Thus, a certain proportion of single CTCs could be those dissociated from clusters composed of clone H. All the single and clustered BM-DTCs detected in the mice orthotopically injected with clone H alone were negative for Ki-67 (Supplementary Fig. S6b), which indicated that clone H-originated DTCs were also in a dormant state. Collectively, our data suggested that dominant colonization of clone H in BM was attributed to the clone’s capacity to form homotypic CTC clusters.

### Regulation of actomyosin associated with E-Cadherin is crucial for stable cell aggregation under FSS conditions

**Figure 7:**
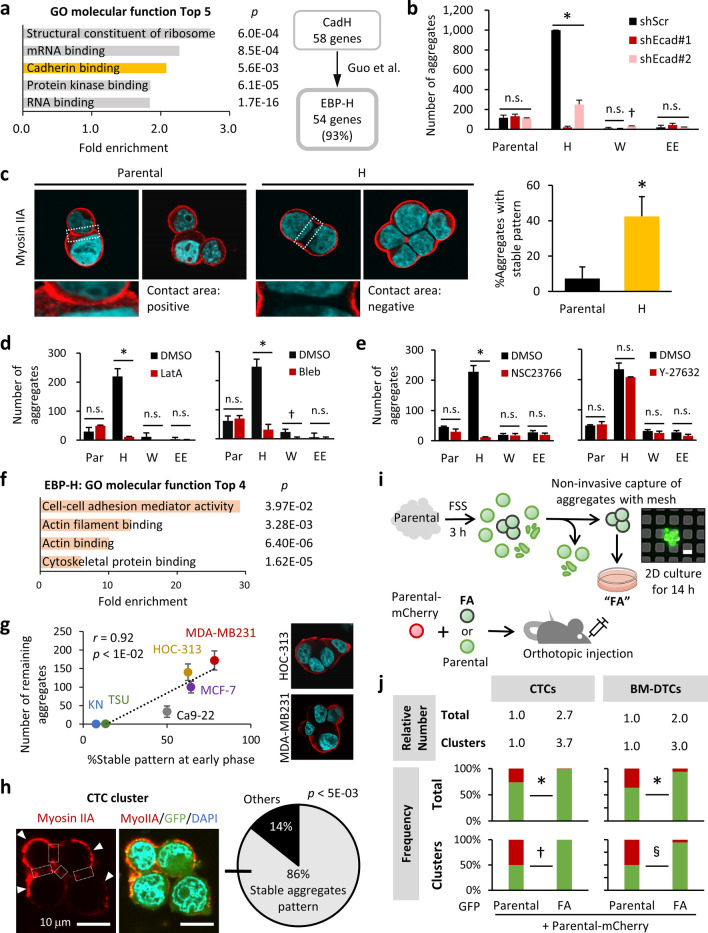
Regulation of actomyosin associated with E-cadherin is crucial for stable cell aggregation in FSS. (**a**) Left panel: GO enrichment of specifically up-regulated genes in clone H. The *p*-values represent Fisher's exact test with Bonferroni correction results. Right panel: CadH genes overlapped with reported EBP genes were defined as EBP-H. (**b**) The number of aggregates from the indicated cells transduced with scramble shRNA (shScr), or shRNA targeting E-cadherin (shEcad) after 3 h of suspension culture under FSS conditions. Values are means ± SEM of triplicate samples. *p < 0.0001; ^†^*p* < 0.005 (two-tailed t tests). (**c**) Results of myosin IIA immunofluorescence staining of cell aggregates at 1 h of suspension culture under FSS conditions. Representative images of cell aggregates are shown (left panels). The regions surrounded by white dotted lines are magnified in the adjacent images. Frequency of cell aggregates showing “stable aggregates pattern” are shown (right panel). *p < 0.01 (two-tailed t tests). (**d**,**e**) Number of aggregates generated by SAS parental (Par) and the clones pretreated with LatA (**d**, left panel), Bleb (**d**, right panel), 50 μM NSC23766 (**e**, left panel), or 10 μM Y-27632 (**e**, right panel) at 3 h of suspension culture under FSS conditions. Values are means ± SEM of triplicate samples. *p < 0.0001; ^†^*p* < 0.005 (two-tailed t tests). (**f**) GO enrichment of EBP-H signature genes. The *p*-values represent Fisher's exact test with Bonferroni correction results. (**g**) Correlation between the frequency of aggregates with “stable aggregates pattern” (myosin IIA distribution) at early phase (30 min) (X-axis) and the number of aggregates detected at 12-h suspension culture in FSS (Y-axis) (left panel). Representative images of aggregates with “stable aggregates pattern” in MDA-MB231 and HOC-313 are shown (right panels). (**h**) Results of myosin IIA immunofluorescence staining of CTC clusters derived from the SAS-GL xenografts. Representative images of a CTC cluster (left panels) and average frequency of clusters (right panel) with “stable aggregates pattern” are shown. Arrowheads indicate the myosin IIA (MyoIIA) on the cortex at free external surfaces. Cell–cell contact areas are surrounded by white dotted lines. (**i**) Diagram showing the experimental setup for the selection of FA cells, and subsequent injection of the mixed tumor cells into mice. Inserted picture shows the aggregates captured on the metal sheet filter. Scale bar, 20 μm. (**j**) Proportions of total cells (Total) and cell clusters positive for only GFP (green) or mCherry (red) in CTCs (left panels) and BM-DTCs (right panels) were calculated for each pooled sample. Relative average numbers of total cells and clusters from each mouse are given above each bar. *p < 0.0001; ^†^*p* < 0.0005; ^§^*p* < 0.05 (Pearson's χ^2^ test).

To ascertain the molecular features contributing to the stabilization of aggregates under mechanical stress, we used mRNA sequencing method to analyze global gene expression in clones H and EE under static condition, by comparing with that in the parental population. Our gene ontology (GO) analyses revealed that the term “cadherin binding (GO:0045296)” was significantly enriched in up-regulated genes in clone H (Fig. 7a) but not in clone EE (Supplementary Fig. S7a), and we then defined this gene signature (58 genes) as CadH. Via literature-based analyses, we found that 54 (93%) genes in the CadH signature overlapped with those encoding E-cadherin-binding proteins (EBPs) reported by Guo et al.^24^ (Fig. 7a; Supplementary Table S1). This gene set was defined as EBP-H.

This GO analysis data led us to assess the involvement of E-cadherin in the stable cell aggregation in FSS. Although clone H did not demonstrate higher E-cadherin level than the parental cells and clones W and EE (Supplementary Fig. S7b), suppression of E-cadherin expression by shRNA dramatically reduced aggregate numbers in clone H at 3 h of suspension culture (Fig. 7b; Supplementary Fig. S7c). In contrast, at this point, no apparent decrease of aggregates was observed in the parental population-, clone W-, and clone EE-derived unstable aggregates, most of which would eventually undergo dissociation. Thus, intercellular homophilic adhesion of E-cadherin was essential to generate and maintain cell–cell adhesion in clone H under FSS conditions. Our data indicated that this E-cadherin-mediated stable adhesion depended primarily on factors such as its binding components rather than the abundance of E-cadherin itself, as supported by the upregulation of EBPs. Actually, the adhesion energy of cadherin bonds is generally too low to mediate cell–cell adhesion even under static conditions^25–27^. Therefore, E-cadherin by itself is still insufficient to fully explain the stability of cell aggregates under FSS conditions. Instead, a large body of evidence shows that regulation of cortical tension by the filamentous actin (F-actin)-myosin machinery (actomyosin) is critical for stabilizing cadherin-based cell–cell adhesion^28,29^. Cortical tension basically shrinks the contact area, so reduced cortical tension primarily due to downregulation of non-muscle myosin II at the contact area will stabilize attachment^27^. Also, in cell aggregates or sheets, high cortical tension at the surface relative to lower tension at internal cell–cell borders should be maintained to promote adhesion^30^. Mechanistically, finely regulated F-actin turnover that is controlled by myosin contractility is essential for such mechanical polarization and hence stable cell–cell adhesion via E-cadherin^31–33^.

We thus assessed the tension applied to cells in aggregates at an earlier time point (FSS for 1 h), before cell aggregates dissociate. Consistent with the data described above, in nearly half of the clone H-derived aggregates, myosin IIA was rarely observed on cell–cell contacts but instead was apparent on the cortex at the free external surfaces (Fig. 7c). This myosin IIA distribution was defined as “stable aggregates pattern”. Only 7% of the aggregates from parental cells showed such distribution, however (Fig. 7c, right panel), that is, most aggregates showed a strong myosin IIA signal along all the attachment surfaces continuous to the free surface cortex (Fig. 7c, left panel). Consistent with the case of E-cadherin knockdown, pretreatment with actin polymerization inhibitor, LatA^33,34^ (Fig. 7d, left panel), or myosin II inhibitor, Bleb^35^ (Fig. 7d, right panel) strongly reduced cell aggregation in clone H under FSS conditions without reducing cell viability prior to FSS exposure (Supplementary Fig. S7d), but not in the parental population and clone EE. Clone W exhibited dependency on myosin activity to a certain extent (Fig. 7d, right panel), which was however not enough for efficient cell aggregation. Indeed, pretreatment with these inhibitors for a short period (1 h) reduced the number of clone H-derived aggregates with stable pattern to the same level as other clones and the parental cells (Supplementary Fig. S7e). These data revealed that regulation of cortical actomyosin dynamics was required for the formation of “stable aggregates pattern”, and thus stable cell–cell adhesion under FSS condition.

The Rho family GTPases are important regulators of actin redistribution, in which Rac1, but not Rock, can stabilize adherens junctions by promoting the formation of cortical actin bundles^36,37^. Furthermore, FSS promotes cadherin-based cell–cell adhesion and thus elevate barrier function of endothelial cells via Rac1 activation by the guanine-exchange factor (GEF) Trio^38^. NSC23766 prevents Rac1 activation by Rac-specific GEFs without inhibiting Cdc42 or RhoA activation^39^. In clone H, similar to the cases of LatA and Bleb, pretreatment with NSC23766 led to inefficient formation of stable aggregates in FSS (Supplementary Fig. S7f, left panel) that resulted in the reduction of aggregates number in a dose-dependent manner (Fig. 7e, left panel; Supplementary Fig. S7g), whereas a selective Rock inhibitor Y-27632^40^ did not (Fig. 7e, right panel; Supplementary Fig. S7f, right panel), even at a higher concentration enough for inducing cell morphological changes; i.e., projection of long protrusions from cell bodies^41,42^ (Supplementary Fig. S7h). Notably, we observed no apparent effect of both inhibitors on cell viability prior to FSS exposure (Supplementary Fig. S7i). Collectively, these results suggested that optimization of cortical actomyosin under FSS conditions was dependent on Rac1 activation.

Expression of structural and regulatory components of the actin cytoskeleton and contractile system affects actomyosin remodeling. Their transcription is coordinated in a context-dependent manner^43^. Consistent with the observed dependency on actomyosin turnover, our transcriptome analyses also revealed the enrichment of the GO term “actin cytoskeleton organization (ACO; GO:0030036)” (35 genes) and the descendants in specifically up-regulated genes in clone H (Supplementary Fig. S7j; Supplementary Table S2) but not in clone EE (Supplementary Table S3). This list of genes, 69% of which encode actin-binding proteins, consisted of important regulators of actin dynamics, with some overlapping functions (Supplementary Table S4), such as acting on regulation of polymerization, bundle assembly, and polarity of actin filament (AF), and Rho signaling. In addition, genes involved in actomyosin organization and maintenance were included (“Others” in Supplementary Fig. S7j). Importantly, the EBP-H signature was also primarily characterized by actin cytoskeleton-binding factors as well as cell–cell adhesion mediators (Fig. 7f; Supplementary Table S5). Accordingly, the top-ranked biological processes in EBP-H included AF capping and regulation of AF polymerization (Supplementary Fig. S7k; Supplementary Table S1 and Supplementary Table S5). Collectively, our data from SAS-GL cells imply that preeminent capacity to generate FSS-adaptive cell aggregates is at least partly owing to the higher basal expression of actomyosin regulators, especially those bound to E-cadherin.

To determine whether difference in the cell intrinsic capacity of actomyosin regulation generally affect mechanical adaptation of cell aggregates, we examined other human cell lines derived from HNSCC (TSU, Ca9-22, KN, and HOC-313) and breast cancer (MCF-7 and MDA-MB231). Consistent with the case of SAS-GL cells, frequency of “stable aggregates pattern” at early phase (30 min) significantly correlated with the number of viable aggregates detected after relatively long-term (12 h) suspension culture in FSS (Fig. 7g). Therefore, successful actomyosin regulation generally accounts for the stability of tumor cell aggregates in FSS, and frequency of stable aggregates may depend on the content of clones having such property within each heterogeneous population. Importantly, most of the detectable CTC clusters derived from the orthotopically injected heterogeneous SAS-GL showed “stable aggregates pattern” (Fig. 7h), indicating that regulation of cortical tension polarization is crucial for CTC clusters to travel in the bloodstream.

On the basis of these observations, we hypothesized that certain tumor cell population capable of forming FSS-adaptive aggregates resistant to anoikis could efficiently generate clustered CTCs with high ability for distant organ colonization. To verify this hypothesis, we selected potentially CTC cluster-originating cell populations by subjecting SAS-GL parental cells to 3-h suspension culture under FSS condition in a serum-free medium, followed by non-invasive capture of generated cell aggregates with a metal sheet filter (CELLNETTA) (Fig. 7i; Supplementary Fig. S7l). Almost only aggregates were captured on the 20-μm mesh (see “Methods”). With the purpose of removing potentially damaged cells within aggregates, we seeded the recovered aggregates on regular culture dish overnight under static condition. By analyzing a part of the captured aggregates, we confirmed that 96.8% of them showed “stable aggregates pattern” (Supplementary Fig. S7m). Consequently, most cells reattached on the dish bottom, and thereafter the cells within each aggregate adhered to each other while maintaining epithelial morphology (Supplementary Fig. S6n). We designated this bulk cell population derived from FSS-adaptive aggregates as FA cells (Fig. 7i). We mixed the GFP-expressing FA cells or parental cells with an equal amount of mCherry-expressing parental cells, and then orthotopically injected them into mice. In the primary tumors, there was no obvious increase in the area occupied by FA compared to parental GFP-expressing cells (Supplementary Fig. S6o). Despite that, in the mixture containing FA cells, the total numbers of CTCs and BM-DTCs, both of which were primarily occupied by FA cells, significantly increased (Fig. 7j). The numbers of clusters in CTCs and BM-DTCs were also higher in the mice injected with FA, and most of these clusters were occupied by only FA cells (Fig. 7j; Supplementary Fig. S6p). These trends observed were similar to clone H-mixed tumors, suggesting that tumor cells with high ability to form FSS-adaptive aggregates dominantly colonize distant organs via generating homotypic CTC clusters.

### Clinical significance of the gene expression signatures in the CTC cluster-originating clone

**Figure 8:**
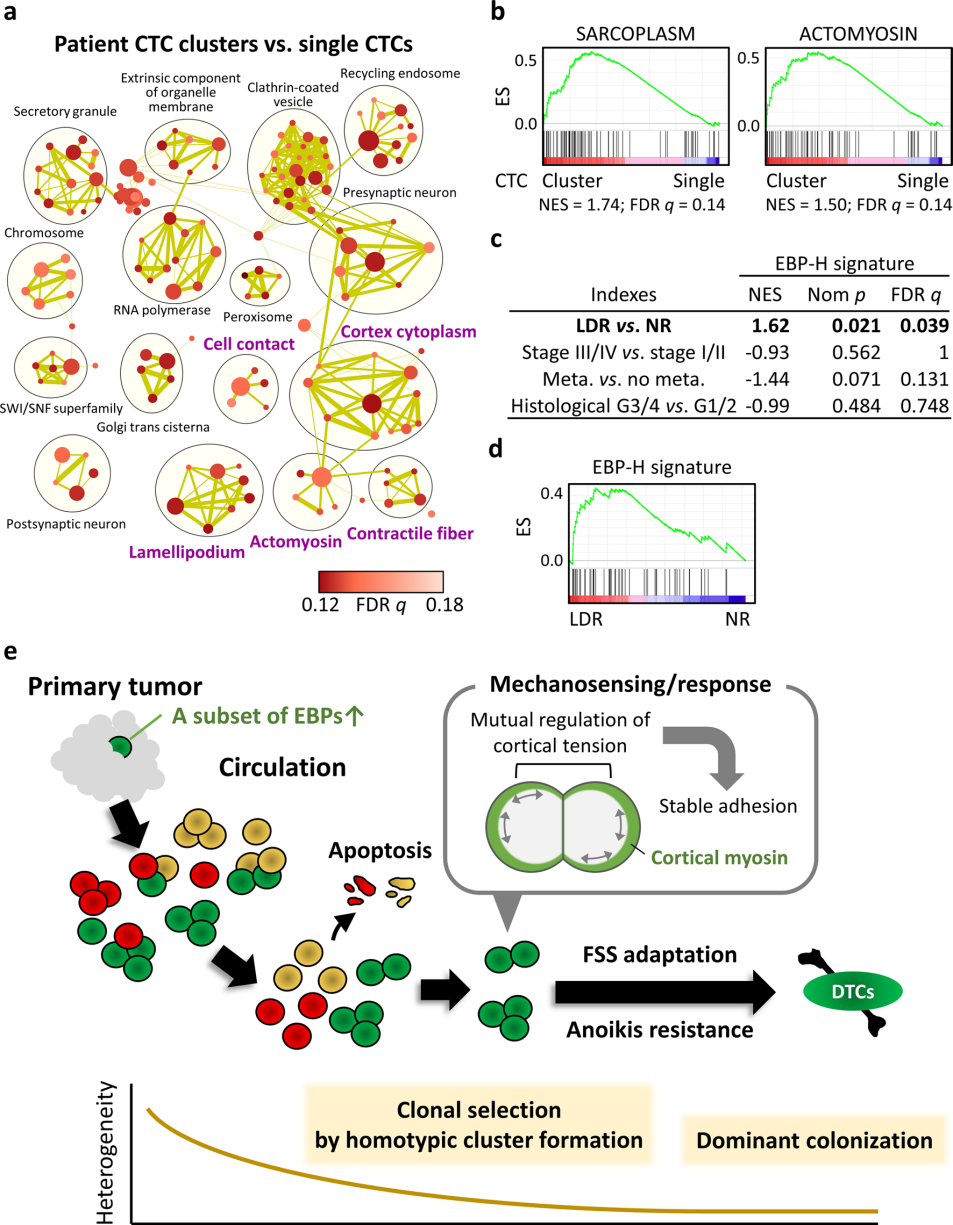
Clinical significance of gene expression signatures in the CTC cluster-originating clone. (**a**) GSEA comparing breast cancer patient-derived CTC clusters with single CTCs, thereby illustrating cellular components that characterize CTC clusters. Cytoscape and Enrichment Map were used to visualize GSEA results as a network of enriched gene sets (FDR *q*-value < 0.18). Nodes representing enriched gene sets are grouped and annotated by their similarity according to related gene sets. The node size is proportional to the number of genes in each gene set. Gene sets are shown with enrichment significance (FDR *q*-value), which is seen as a node color gradient. The proportion of genes shared between gene sets (similarity coefficient) is represented as the thickness of green lines between nodes. Black circles represent summarized gene set clusters based on AutoAnnotate. The annotation names involved in cortical actin-myosin assemblies and cell–cell adhesion are in purple type. Detailed GSEA results can be found in Supplementary Table S6. (**b**) GSEA enrichment plots showing representative enriched gene sets in up-regulated genes in CTC clusters compared with single CTCs. Normalized enrichment scores (NESs) and FDR *q*-values are shown for sarcoplasm and actomyosin. (**c**) GSEA results for genes in the signature EBP-H for various clinicopathological indexes in the TCGA HNSCC cohorts of primary tumor samples. NES, nominal (Nom) *p*-values, and FDR *q*-values are shown. Meta., metastasis. (**d**) GSEA enrichment plots for the LDR group compared with the NR group, which correspond to (**c**). (**e**) A proposed model in which relatively rare clones in primary tumors, which highly express a subset of EBP genes, aggressively generate and maintain homotypic CTC clusters through cell–cell adhesion by E-cadherin in blood circulation, which leads to dominant colonization of secondary sites. Homotypic CTC clusters originating from such clones can reorganize the actin cytoskeleton along with regulation of cortical tensions by myosin in response to external FSS, which leads to lower cortical tension along attachment surfaces compared with their free external surfaces and thereby strengthened cell–cell adhesion.

We then analyzed mRNA expression profiles in patient-derived CTC clusters and single CTCs via published breast cancer expression data (GSE111065)^44^, in view of the fact that the mechanical force in blood circulation is generally independent of types and origins of cancer. Our gene set enrichment analysis (GSEA)^45,46^ revealed that CTC clusters were mostly characterized by gene sets involved in cortical actin-myosin assemblies (Fig. 8a,b; Supplementary Table S6). Remarkably, sarcoplasm (GO:0016528), related to genes encoding muscle cell cytoplasmic components, was the most enriched term of all the C5 cellular component gene sets (*n* = 1001) among the up-regulated genes in CTC clusters (Fig. 8b). Comparable results were obtained when the dataset for mouse xenografts transplanted with cell lines established from the corresponding primary tumors was analyzed (Supplementary Fig. S8). Thus, CTC clusters in the bloodstream are enriched with tumor cells highly expressing genes that are involved in actomyosin regulation at the cell cortex, which agreed with the findings derived from the HNSCC subpopulation we identified.

Finally, we investigated the clinical significance of the intrinsic gene expression signature of an identified CTC cluster-originating clone by using the HNSCC dataset from The Cancer Genome Atlas (TCGA)^47,48^. As a noteworthy result, our GSEA revealed a significant enrichment of the EBP-H signature among the genes up-regulated in primary HNSCC tumors of patients who manifested locoregional and distant recurrence (LDR) compared with patients who had no recurrence (NR) (Fig. 8c,d). This gene signature was associated with the absence of metastasis (pN or pM) at the initial surgical treatment, although the statistical significance was relatively low (Fig. 8c). We did not find any association of the gene expression signature with pathological stage or histological grade, nor did we find any gene set with a significant association with LDR or NR in the GSEA querying C5 cellular component. These data suggest a contribution of EBP-H, expression of which likely reflect the content of the CTC cluster- and thus latent DTC-originating clones, to the progressive dormant phenotype, as if to reflect our original xenograft model of tumor dormancy.

Taken together, we thus demonstrate one of the functional clonality of CTC clusters which contributes to distant colonization. Our data suggest a model in which a subset of clones overexpressing EBPs primarily consisting of actin-binding proteins within primary tumors preferentially generate homotypic anoikis-resistant clusters in the bloodstream, which inevitably leads to clonal selection that results in their dominant colonization of secondary sites (Fig. 8e).

## Discussion

Successful colonization of distant organs by CTCs is a critical step that precedes metastatic growth. However, what properties of tumor cells are critical for tumor dissemination and distant colonization have remained unclear. Here, by using high-resolution clonal tracking, we found that only limited numbers of clones in primary populations efficiently circulated in the bloodstream and colonized distant organs. Our data showed that homotypic CTC clustering by specific clones was a principal mechanism responsible for this clonal behavior.

CTC clusters, even though they are rare, are significantly more metastatic than individual CTCs^8^. Because CTCs are rare^8,9^, experimental analysis of CTC clusters is difficult and hence the functional clonality of CTC clusters has continued to be totally unknown. Our analyses with defined sets of clones found a single subclonal line as a CTC cluster-originating clone, which efficiently adapted to distant organs. Consistent with clonal tracking data, the clusters remained almost homotypic even within the original heterogeneous population, which confirmed not only the unique property of this clone but also the importance of sharing equivalent properties within each cluster to maintain cell–cell adhesion under FSS conditions, presenting the possibility that CTC clusters are functionally monoclonal.

As a conceivable mechanism for the clonal composition in CTC clusters, we identified the resistance of individual cells in clusters to FSS. As an important fact, solid cancer cells, without regard to type or origin, are exposed to mechanical forces (FSS) in the bloodstream, and they must withstand or adapt to an applied load to reach target organs. We currently identified E-cadherin as an indispensable junctional protein for stable cell aggregation. This finding is supported by recent observations that E-cadherin is required for metastasis^49,50^ and is expressed in CTC clusters^10^. However, in agreement with the fact that the adhesion energy of cadherins is very small^25–27^, we found that optimization of cortical tension by actomyosin governed the stability of cell–cell adhesion in suspension under FSS conditions. The number, size, recruitment, and compositional turnover of E-cadherin clusters at cell–cell junctions largely depend on the turnover of AF bound to E-cadherin, which is controlled by myosin contractility^32,33^. We therefore assume that the increased expression of a subset of EBPs, which are enriched in actin-binding proteins and actin dynamics regulators, contributes to the maintenance of E-cadherin-based cell–cell adhesion under FSS conditions, i.e., only a small fraction of cancer cells may benefit from E-cadherin bonds at least after entering circulation. Notably, E-cadherin coupled with actomyosin in neighboring cells also serves as an active mechanical integrator in dynamics of cell pairs and tissues^32^. Therefore, a set of E-cadherin molecules and a higher amount of EBPs may be chosen as optimal components of CTC clusters.

When cells are exposed to external mechanical forces, F-actin collapses and then reorganizes with myosin to regulate cortical tension according to applied loads^51^. Given the complexity of the cell cortex^52^, multiple factors rather than one key component likely act together to maintain cell–cell adhesion under conditions of continuous mechanical stress. Intriguingly, we found that CTC cluster-originating clone and patient-derived CTC clusters were intrinsically characterized by higher expression of a broad range of actin/actomyosin dynamics regulators including those binding to E-cadherin. These findings may represent the outstanding processing capability of the cells composing CTC clusters. Importantly, CTC clusters successfully traversed capillary-sized vessels by rapid and reversible unfolding into a chain of single cells via regulation of intercellular adhesions^53^. Therefore, although further studies are needed to show the involvement of mechanosensors, we assume that CTC clusters are not cohesive resistive units but are instead individual mechanosensitive cells in series, so that they have the flexibility to respond to various biophysical stresses during the dissemination processes.

One important issue is how CTC clusters can withstand FSS. Although various mechanisms for anoikis resistance in tumor cells including CTC clusters have been discussed^6^, how CTC clusters acquire a survival advantage remains undetermined. Here, we found that resistance to anoikis was induced by stable aggregation only under FSS conditions, independently of the type of originating cells. Therefore, external mechanical force may coordinate the stable cell–cell adhesion machinery to activate certain common pro-survival strategies. Basically, cytoskeletal tension functions as a second messenger for mechanical signals under the influence of adhesion forces, which regulates cell proliferation and differentiation^54,55^. Patient-derived clustered CTCs have been shown to possess stem-cell-like molecular features compared with single CTCs via altered intracellular signal activation^12^ or epigenetic reprogramming^44^. Indeed, external mechanical forces regulate cell differentiation by using actomyosin machinery^55,56^. Addressing whether mechanotransduction is involved in the prometastatic property of CTC clusters would be interesting.

Our findings including those from clinical dataset suggest that tumor cells that preferentially form CTC clusters and hence dominate DTCs are intrinsically preselected in primary tumors. Complete epithelial-mesenchymal transition (cEMT), which results in loss of E-cadherin expression and cell–cell adhesion, induces solitary tumor cell dissemination, while mesenchymal-epithelial transition, the reversion of EMT, is recognized to be essential for distant colonization^57^. Unlike these binary system, partial EMT produces carcinoma cells in a hybrid epithelial-mesenchymal (E/M) state, inducing collective migration via maintenance of E-cadherin expression^58^. This process is proposed as one mechanism for generating CTC clusters^58,59^. Indeed, single-cell RNA sequencing revealed that the hybrid E/M subpopulation localizes at the leading edge of HNSCC primary tumors, and the hybrid E/M subtype is an independent predictor of adverse clinicopathological features in HNSCC^60^. Consistent with this concept, our data suggested that CTC cluster-originating tumor cells preserved epithelial traits without cEMT, at least partially. Further analyses are needed to determine whether the observed correlation between the expression signature characterizing the CTC cluster-originating tumor cells and increased LDR in HNSCC patients reflects the content of CTC cluster-originating populations, CTC cluster number, and the cells with hybrid E/M state. Given that collective migration is regulated by actomyosin contractility and mechanical forces^61^, it is also worth investigating whether the E/M state determines mechanical response. Regardless of E/M state, it should also be noted that individual tumor cells can aggregate in the blood vessels to promote metastasis^12^.

In conclusion, we used a xenograft model that reproduced HNSCC metastatic dormancy to demonstrate systemic clonal composition in latent DTCs and to show that specific clones in a primary tumor cell population can dominate CTCs by stable homotypic clustering, as a novel clonal selection mechanism leading to selective colonization of distant organs. Our findings emphasize the importance of targeting CTC clusters^44^ and of obtaining a better understanding of tumor cell mechanosensitivity in the metastatic cascade. To this end, investigating whether expression of the gene sets we identified is controlled by a certain master regulator or genomic alterations would be valuable. Additional studies may contribute to the discovery of novel therapeutic strategies useful for targeting and preventing metastatic recurrence.

## Supplementary Information

Supplementary Information.

Supplementary Table S1.

Supplementary Table S2.

Supplementary Table S3.

Supplementary Table S4.

Supplementary Table S5.

Supplementary Table S6.

## Data Availability

Our mRNA-sequencing data and the datasets analyzed during the current study are available through the Gene Expression Omnibus with accession number GSE158933 and GSE111065, respectively. All other data supporting the findings of this study are available within the paper and Supplementary Information.
