# Supplementary material for: Colonization of distant organs by tumor cells generating circulating homotypic clusters adaptive to fluid shear stress
Source: Sci Rep. 2021 Mar 17;11:6150. doi: 10.1038/s41598-021-85743-z (PMC7969766; doi:10.1038/s41598-021-85743-z)
Supplement: Supplementary file 1 — Supplementary Information. [file 41598_2021_85743_MOESM1_ESM.pdf]

Supplementary Information

Colonization of distant organs by tumor cells generating circulating homotypic clusters adaptive to fluid shear stress

Manabu Maeshiro*, Satoru Shinriki*,**, Rin Liu, Yutaka Nakachi, Yoshihiro Komohara, Yukio Fujiwara, Kazuaki Ohtsubo, Ryoji Yoshida, Kazuya Iwamoto, Hideki Nakayama, Hirotaka Matsui**

*Co-first authors of this manuscript.

**Corresponding authors: satorus@kuh.kumamoto-u.ac.jp; hmatsui@kumamoto-u.ac.jp

Supplementary Figure S1

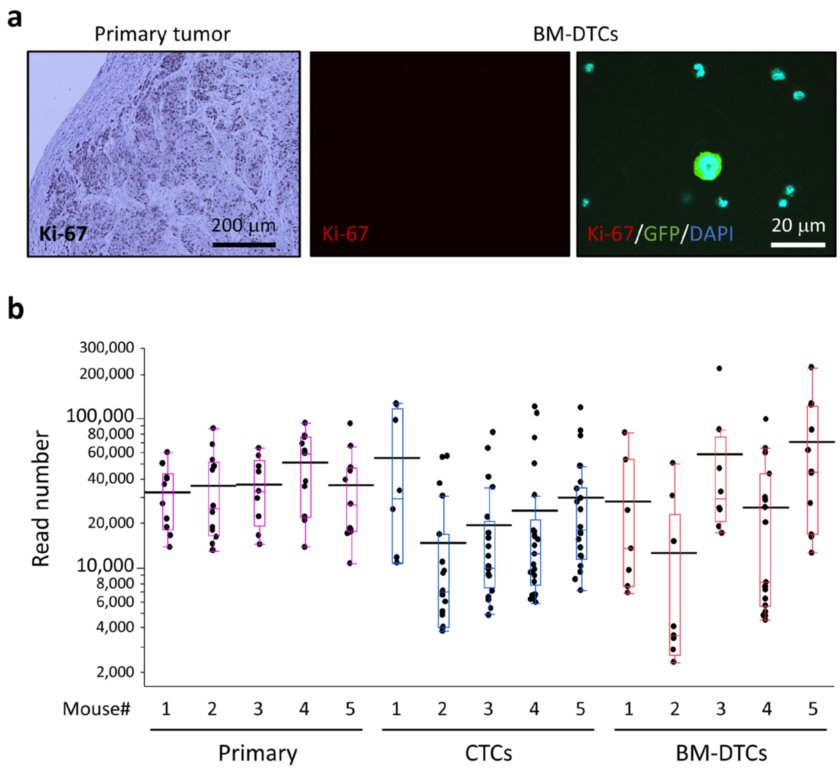

Supplementary Figure S1. Results of Ki-67 staining of BM-DTCs, and sequencing coverage of dominant clones in each sample. a, Results of Ki-67 staining of the primary tumors (left panel) and BM-DTCs (right panels) from the mice injected with SAS-GL cells. Representative images are shown. **b,** Sequencing coverage of dominant clones in each sample is shown. Black horizontal line on each box plot represent the average.

Supplementary Figure S2

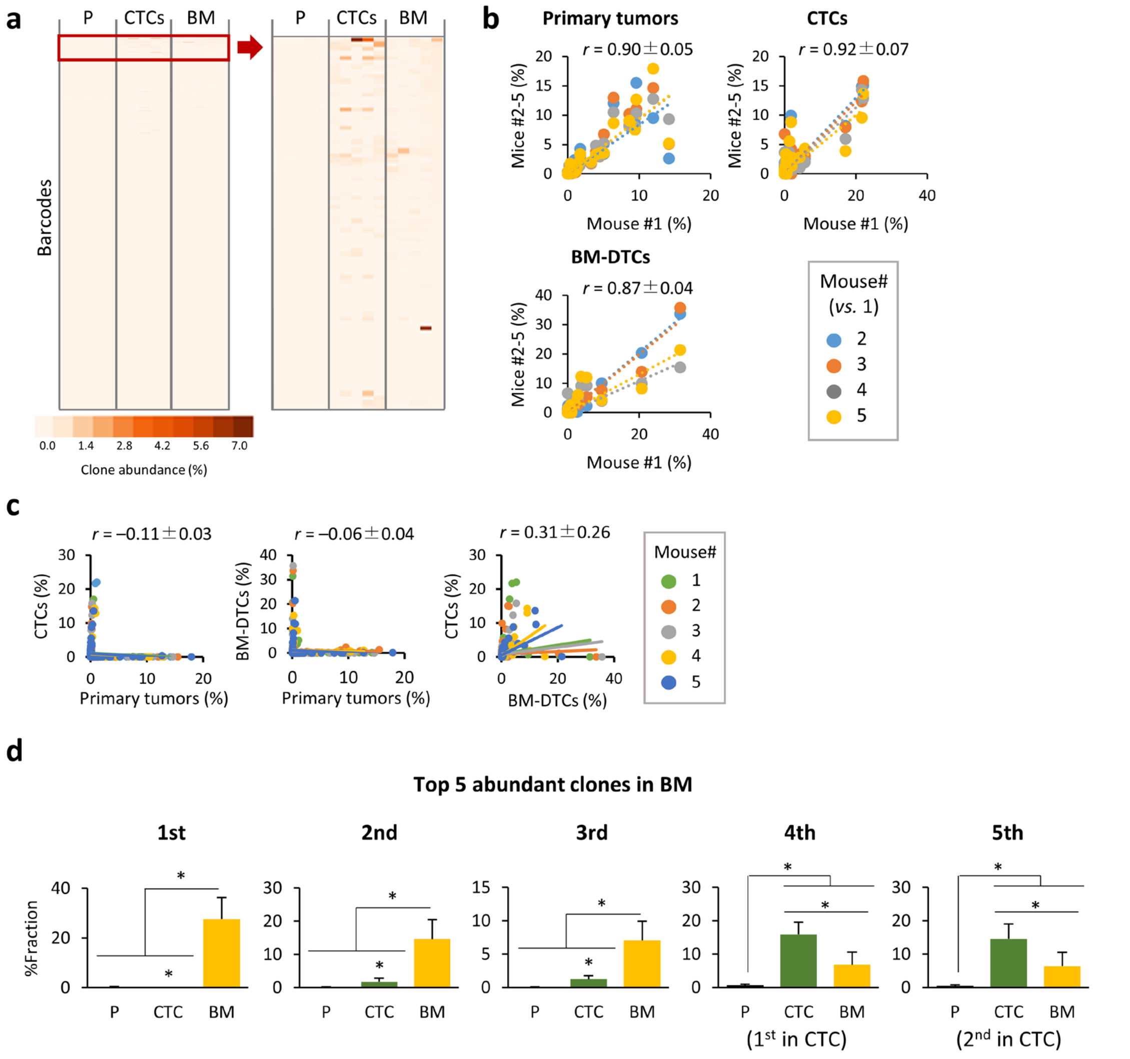

Supplementary Figure S2. Clonal analysis of SAS transplantation by molecular barcoding. a, Relative proportions of clones that did not engraft at all primary sites (P) across all five mice. BM, BM-DTCs. **b,** Correlation of the abundance of unique seeding clones between the mice in each lesion. Clones that engrafted at primary sites are shown (n = 5 mice). Each color dot represents an individual mouse. **c,** Correlations of the abundance of unique seeding clones in each lesion. Clones that engrafted at primary sites in all mice are shown (*n* = 5 mice). Each color represents one sample from an individual mouse. **d,** The abundance ratio of the top five most abundant clones in BM-DTCs. These clones also included the abundant clones in CTCs as shown under the graphs. P, primary tumors; BM, BM-DTCs.

Supplementary Figure S3

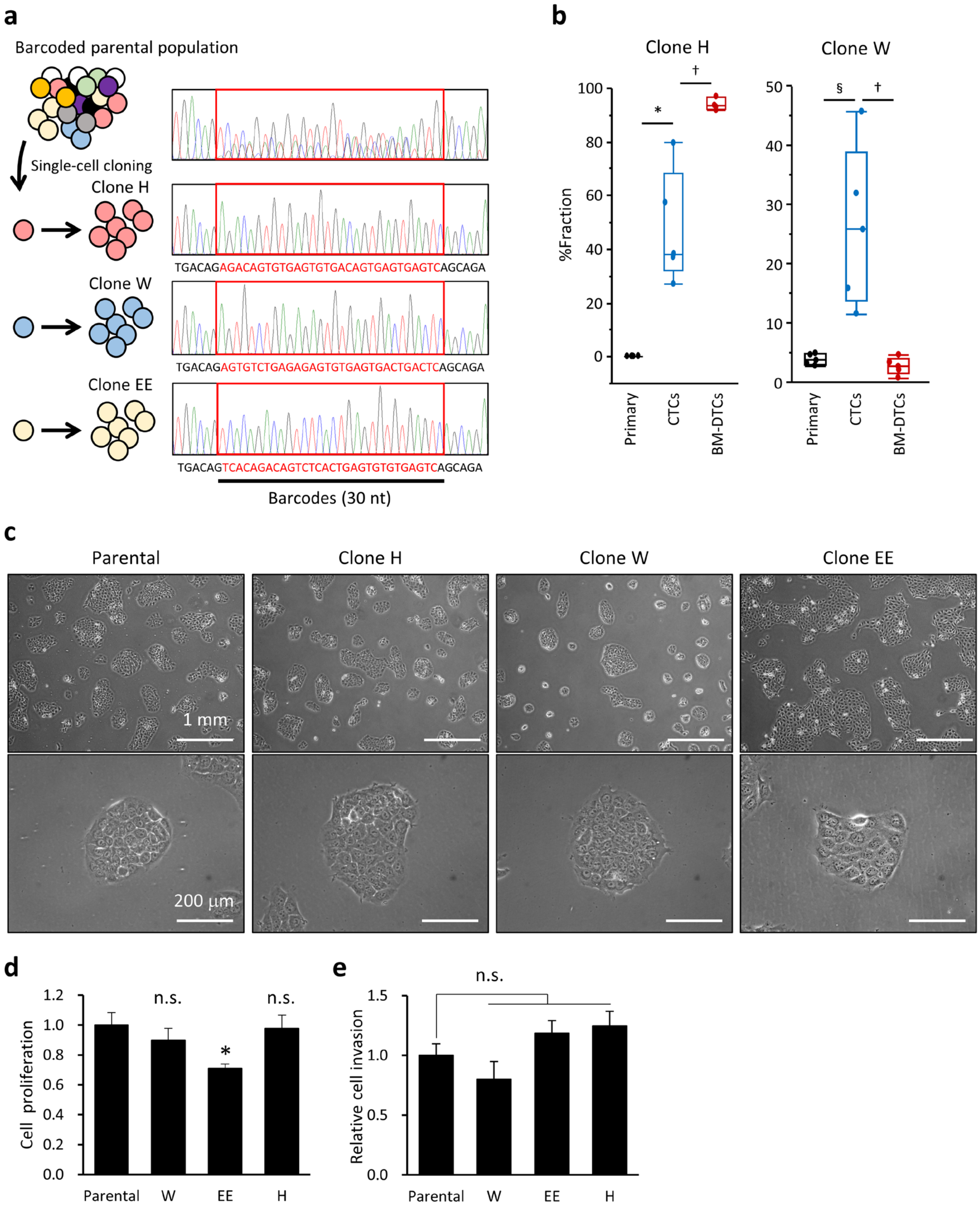

Supplementary Figure S3. Systemic distribution in mice and cellular phenotypic characteristics under static conditions of SAS-derived clones. a, Results of Sanger sequencing of a barcoded parental population, as well as representative subclones (W, EE, and H) established after single-cell sorting. Areas enclosed by red lines indicate barcode sequences (30 nt). Note that each subclone but not the parental population before sorting has a unique barcode sequence. **b,** The abundance of each clone in primary tumors, CTCs, and BM-DTCs is shown. **p* < 0.001; †*p* < 0.005; §*p* < 0.01 (two-tailed t tests). Data correspond to Fig. 3c. **c,** Morphology of parental cells and clones H, W, and EE. Scale bars: 1 mm (upper panels) and 200 μm (lower panels). **d,** Cell proliferation capacity. Samples of 5×10^4 cells were suspended in RPMI 1640 medium containing 10% FBS and then cultured for 3 days. Viable cell numbers were counted. **p* < 0.005; n.s., not significant. **e,** Cells were suspended in serum-free medium on transwell filters coated with Matrigel in the upper chamber, with medium containing 10% serum being used as a chemoattractant in the lower chamber. After 24 h, cell fluorescence was read at 480/520 nm. Values are means ± SEM of triplicate samples.

Supplementary Figure S4

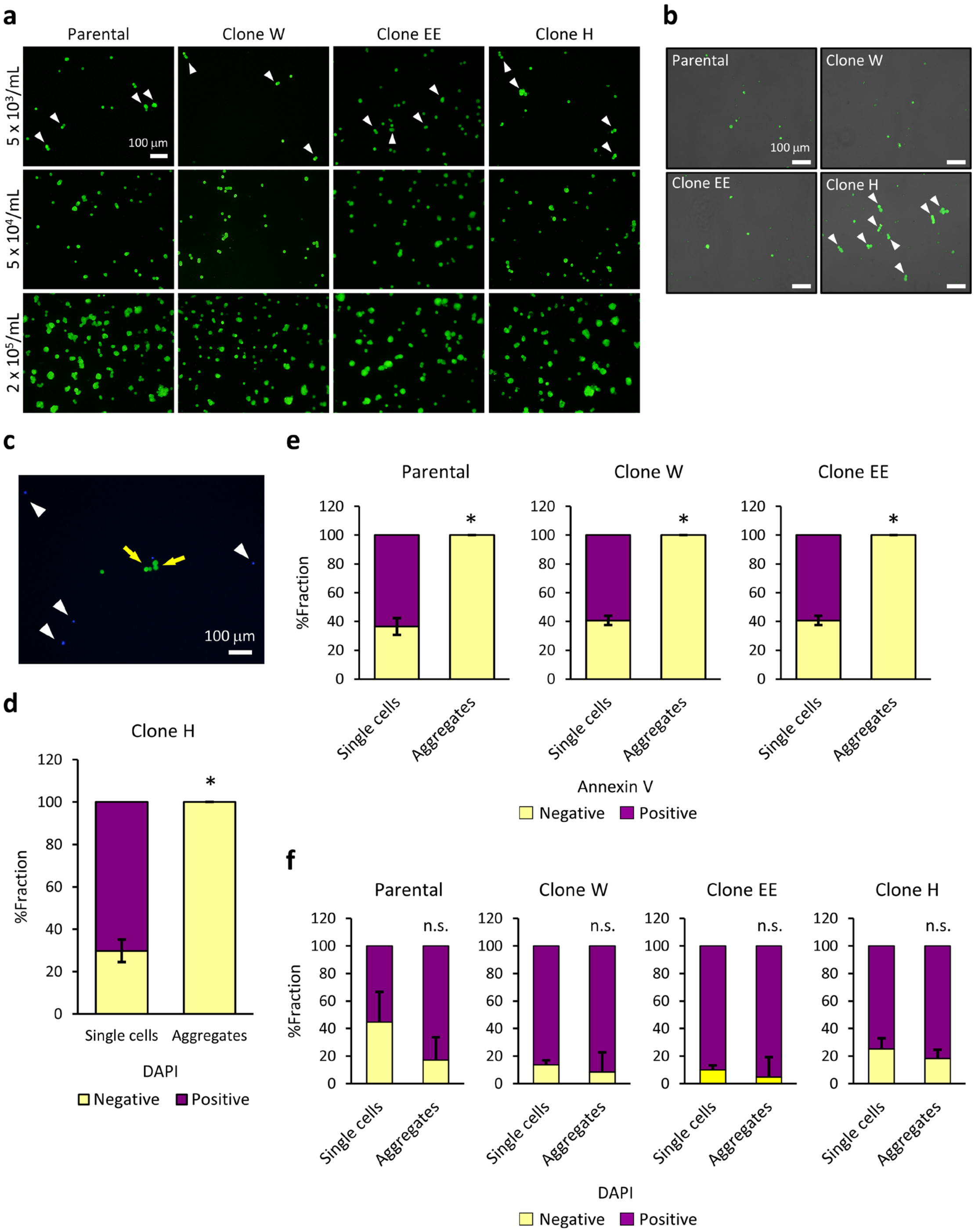

Supplementary Figure S4. Anoikis resistance in cell aggregates generated under FSS conditions. a, Representative images of SAS-GL cells (parental), clone W, clone EE, and clone H at 18 h in the anoikis assay under static conditions. Cells were suspended at the indicated concentrations. Arrowheads indicate cell aggregates. **b,** Representative images of SAS-GL cells (parental), clone W, clone EE, and clone H at 3 h in the anoikis assay under FSS conditions. Arrowheads indicate cell aggregates. **c-e,** Single cells (5×10^3) from SAS-GL cells (parental), clone H, clone W, or clone EE were suspended in serum-free culture medium and rotated at 37 °C for 3 h. Cells were stained with DAPI (**c** and **d**) or annexin V (**d**) and were plated in 96-well plates, after which DAPI- and annexin V-positive results in single cells and aggregates were evaluated. **c,** DAPI staining with clone H cells at 3 h of suspension culture. Arrows and arrowheads indicate DAPI-negative aggregates and DAPI-positive single cells, respectively. **d,** Proportion of DAPI-positive and DAPI-negative cells in single cells and aggregates. *p < 0.0001 (Pearson's χ^2 test). **e,** Proportion of annexin V-positive and annexin V-negative cells in single cells and aggregates. *p < 0.005 (Pearson's χ^2 test). **f,** Proportion of DAPI-positive and DAPI-negative cells in single cells and aggregates under static condition.

Supplementary Figure S5

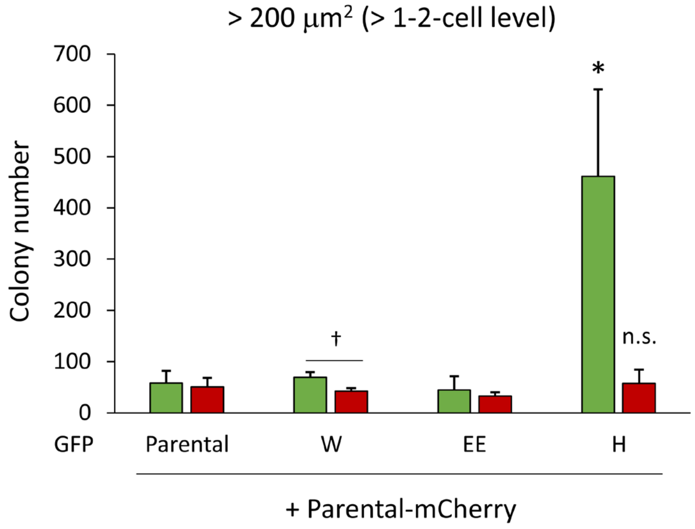

Supplementary Figure S5. Aggressive homotypic colony formation by clone H. Equal numbers of mCherry-expressing parental cells (Parental-mCherry) and GFP-expressing parental cells (Parental), clone W, clone EE, or clone H dissociated into single cells were mixed and suspended in serum-free medium followed by rotation at 37 °C for 22 h. Cells were centrifuged, plated in 6-well plates, and cultured for 6 days. Then, colonies of surviving cells with an area >200 μm^2 were counted. The numbers of GFP-positive colonies and mCherry-positive colonies in each experimental group are shown. Values are means ± SEM of triplicate samples. *p < 0.05 (vs. all other GFP-positive and mCherry-positive cells); $^{\dagger}p$ < 0.05; n.s., vs. mCherry-positive cells in all other groups.

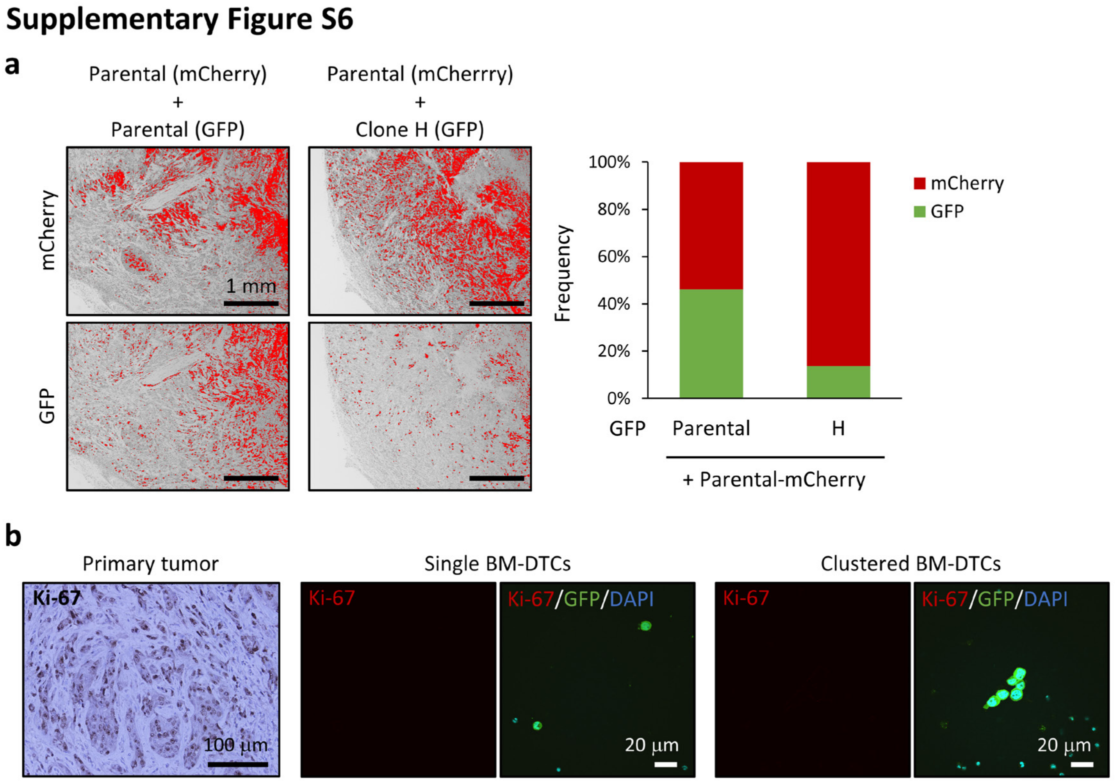

Supplementary Figure S6. Analyses of proportions of tumor cells, and Ki-67 positivity. a, Abundance ratio of tumor cells positive for GFP (parental or clone H) or mCherry (parental) in primary tumors were calculated by using ImageJ software. The images correspond to Fig. 6b, upper panels. **b,** Results of Ki-67 staining of the primary tumors (left panel), single BM-DTCs (middle panels), and clustered BM-DTCs (right panels) from the mice injected with clone H alone. Representative images are shown.

Supplementary Figure S7

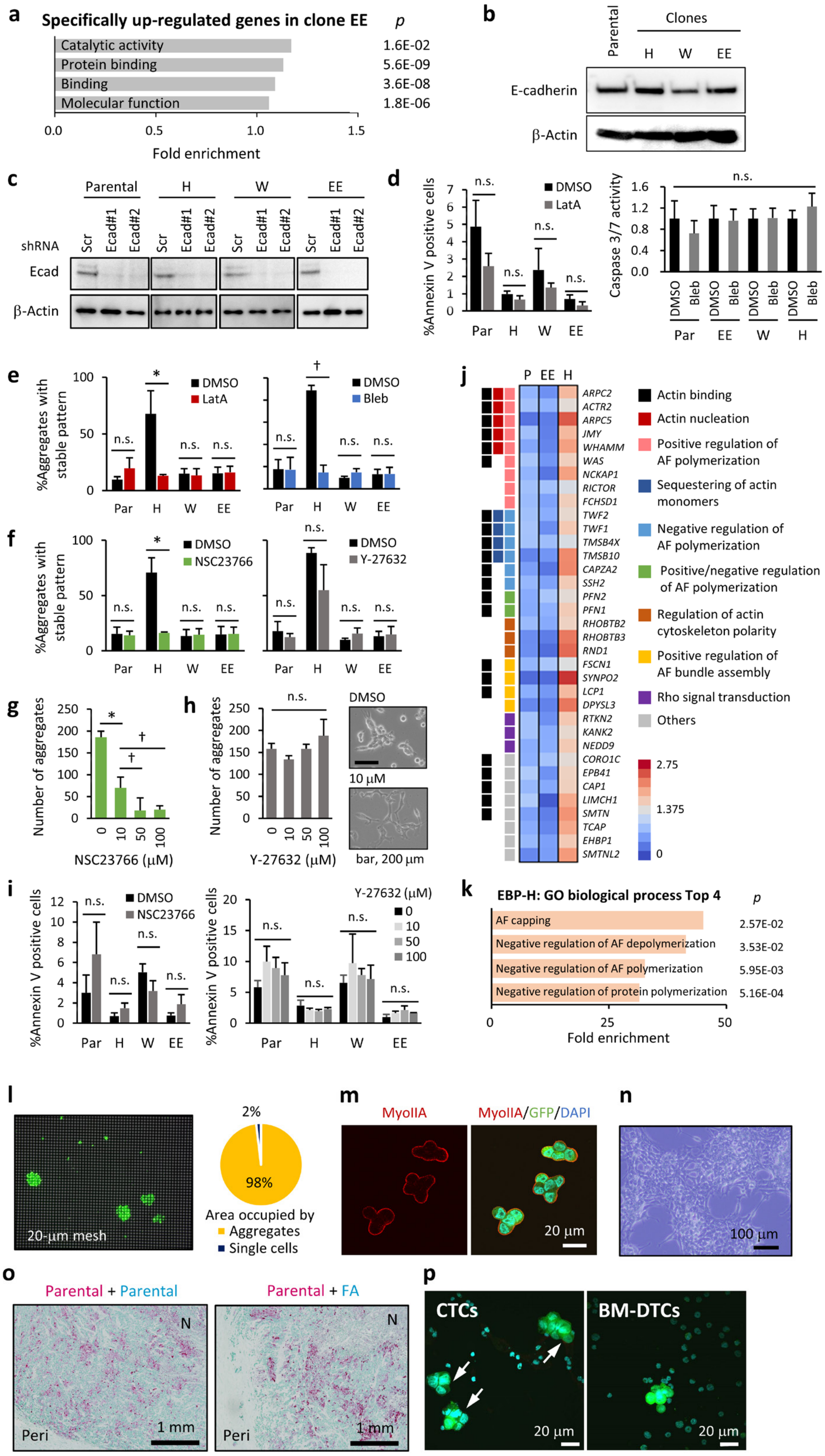

Supplementary Figure S7. Regulation of actomyosin associated with E-cadherin in stable cell aggregation under FSS condition. a, GO enrichment of the up-regulated genes in clone EE. Enriched GO terms identified by GO PANTHER analyses of the "molecular function" category are shown. **b,** Expression of E-cadherin protein was evaluated by using immunoblotting. The results represent three independent experiments. **c,** Expression levels of E-cadherin in SAS-parental and the subclones stably transduced with shScr or shEcad were examined by using immunoblotting. β-Actin was used as a loading control. Uncropped images of blots in **b** and **c** can be found in Supplementary Information. **d,** Percentage of Annexin V-positive cells, and relative caspase 3/7 activity were measured after 3-h treatment with LatA (1 μM, left panel) and Bleb (20 μM, right panel), respectively. **e** and **f,** Results of myosin IIA immunofluorescence staining of cell aggregates at 1 h of suspension culture under FSS conditions. Cells were pretreated with 1 μM LatA (**e**, left panel), 20 μM Bleb (**e**, right panel), 50 μM NSC23766 (**f**, left panel), or 10 μM Y-27632 (**f**, right panel) for 3 h. Frequency of cell aggregates showing "stable aggregates pattern" are shown. Par: parental. $*p < 0.01$; $^{\dagger}p < 0.0001$ (two-tailed t tests). **g,** Number of aggregates generated by SAS parental (Par) and the clones pretreated with the indicated concentrations of NSC23766 at 3 h of suspension culture under FSS conditions. Values are means ± SEM of triplicate samples. $*p < 0.0001$; $^{\dagger}p < 0.01$ (two-tailed t tests). **h,** Left panel: Number of aggregates generated by SAS parental (Par) and the clones pretreated with the indicated concentrations of Y-27632 at 3 h of suspension culture under FSS conditions. Values are means ± SEM of triplicate samples. Right pane: Cell morphology of clone H treated with 10 μM Y-27632 for 3 h. **i,** Percentage of Annexin V-positive cells was measured after 3-h treatment with 10 μM NSC23766 (left panel) and the indicated concentrations of Y-27632 (right panel). **j,** A heat map showing expression of 35 genes included in the GO term ACO enriched in clone H. Associated representative biological processes are shown for each gene. P, parental; EE, clone EE; H, clone H. **k,** The top-ranked biological processes in EBP-H genes included AF capping, negative regulation of AF depolymerization, negative regulation of AF polymerization, and negative regulation of protein polymerization. **l,** Results of performance evaluation of CELLNETTA independently performed using SAS-GL cells. Left panel: Representative image of captured cell aggregates on the 20-μm mesh. Right panel: Abundance ratio of cells from aggregates and single cells on the 20-μm mesh. Area occupied by aggregates or single cells were calculated by using ImageJ software. **m,** Results of myosin IIA immunofluorescence staining of the captured aggregates. Representative images are shown. MyoIIA: myosin IIA. **n,** Representative image of recovered cells from

aggregates. After suspension culture under FSS condition for 3 h, cell aggregates were captured with the 20-μm mesh followed by being seeded on the tissue culture dish. Cells were photographed 14 h after seeding. **o,** Representative images of primary tumors from mixtures of parental/parental cells (left panels) and parental/FA cells (right panels). Peri: peripheral lesion. N: necrotic lesion. **p,** Representative images of clusters in CTCs (left panel) and BM-DCTs (right panel) from mice injected with FA-mixed tumors. Arrows indicate CTC clusters.

Supplementary Figure S8

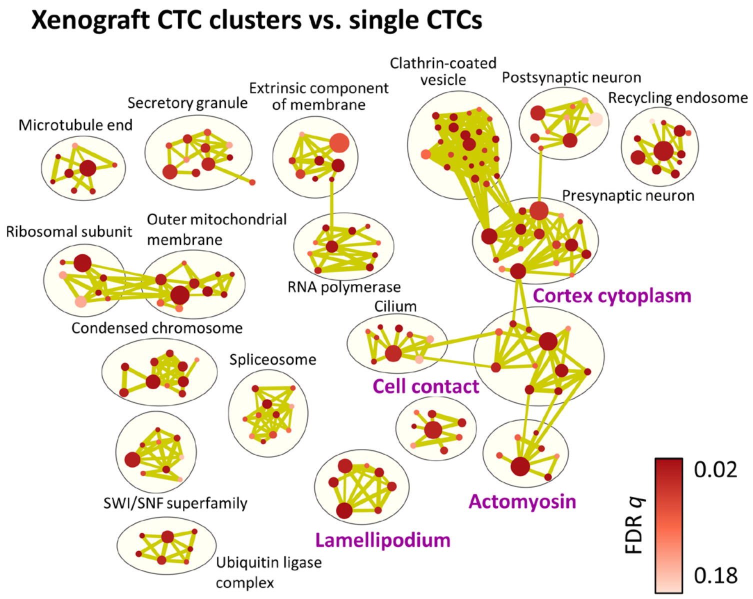

Supplementary Figure S8. GSEA data for xenograft-derived CTC clusters compared with single CTCs. GSEA results comparing CTC clusters with single CTCs derived from mouse xenografts inoculated with breast cancer cell lines established from patients (GSE111065) illustrate cellular components that characterize CTC clusters. Cytoscape and Enrichment Map were used to visualize GSEA results as a network of enriched gene sets (FDR *q*-value < 0.18). Nodes that illustrate enriched gene sets are grouped and annotated by their similarity in agreement with related gene sets. The node size is proportional to the number of genes in each gene set. Gene sets are shown with enrichment significance (FDR *q*-value), which is seen as a node color gradient. The proportion of genes shared between gene sets (similarity coefficient) is represented by the thickness of green lines between nodes. Black circles represent summarized gene set clusters based on AutoAnnotate. The names of the annotations involved in cortical actin-myosin assemblies and cell-cell adhesion are in purple type.

Uncropped images of agarose gels and immunoblots

Figure 1b

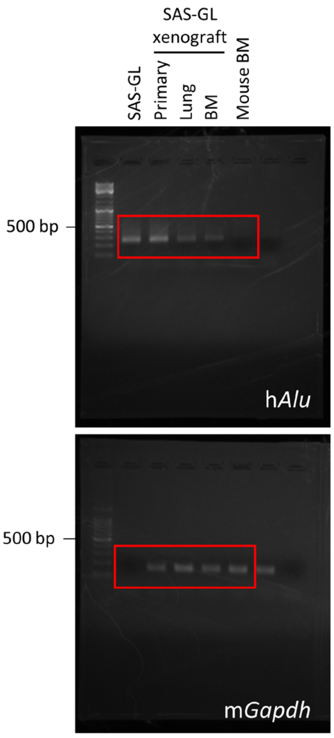

Supplementary Figure S7b

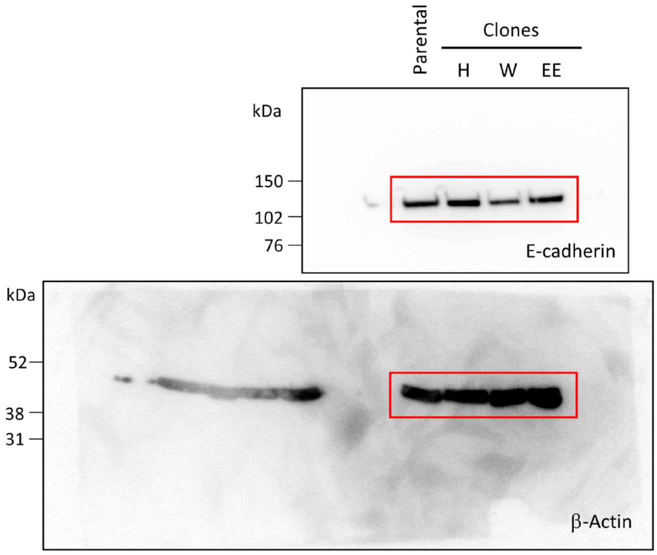

Supplementary Figure S7c

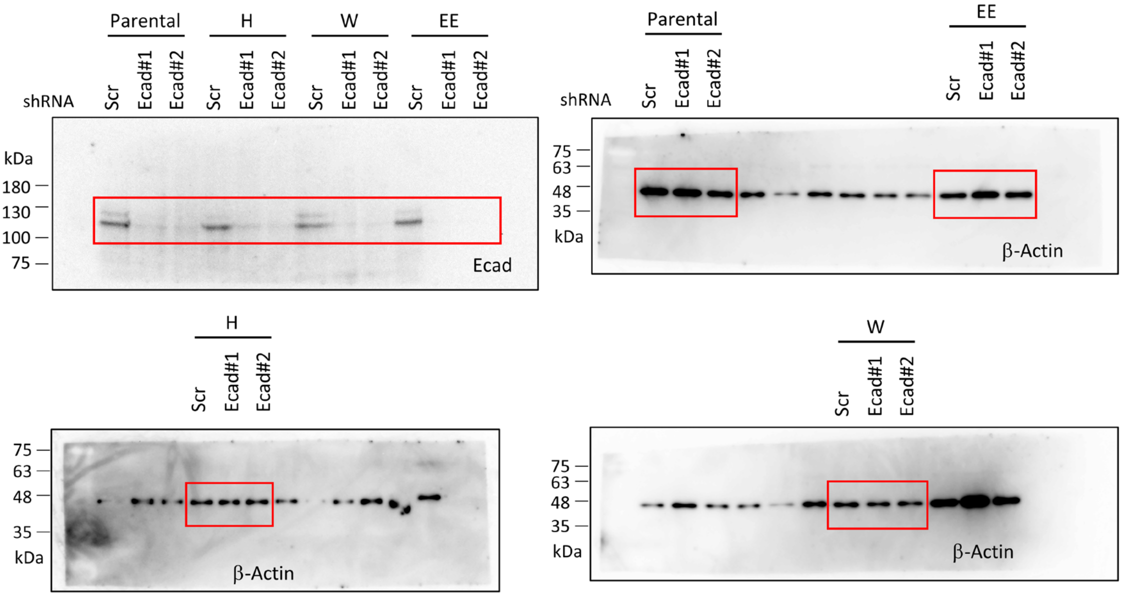

Supplementary Methods

PCR Amplification of the Human *Alu* Sequence. SAS cells in the organs of the mice were detected by PCR amplification of the human *Alu* (h*Alu*) sequence[1,2]. Genomic DNA from mouse tissues prepared as described above was isolated by using NucleoSpin Tissue kits (Takara Bio) according to the manufacturer's instructions. Genomic DNA (20-ng samples) was amplified by using Fast SYBR Green Master Mix (Thermo Fisher Scientific). Primers used were as follows: h*Alu* forward: 5′-ACGCCTGTAATCCCAGCACTT-3′; h*Alu* reverse: ′5′-TCGCCCAGGCTGGAGTGCA-3′. PCR was performed according to the following procedure: denaturation at 95 °C for 20 s, followed by 25 cycles at 95 °C for 3 s and then at 60 °C for 30 s. Amplification of mouse *Gapdh* (m*Gapdh*) was used as an internal control.

Preparation of Sequencing Libraries for Barcode Analyses. The barcode sequences were amplified by using a two-step PCR protocol. For each sample, PCR reactions for 200 ng of genomic DNA were performed with KOD Plus (TOYOBO, Tokyo, Japan). Sequences of primers used were as follows: forward primer: 5′-tcgtcggcagcgtcagatgtgtataagagacagACTGACTGCAGTCTGAGTCTGACAG-3′ and reverse primer: 5′-gtctcgtgggctcggagatgtgtataagagacagCTAGCATAGAGTGCGTAGCTCTGCT-3′. The first PCR was carried out for 30 cycles, and the size of the PCR product was confirmed as 150 bp by using the Agilent 2200 TapeStation D1000 (Agilent Technologies, Palo Alto, CA). PCR products were purified by using Agencourt AMPure XP beads (Beckman Coulter, Pasadena, CA). Then, via the Nextera XT index kit (Illumina, San Diego, CA), indexes were added in an 8-cycle PCR by using KOD Plus (TOYOBO), followed by purification with Agencourt AMpure XP beads (Beckman Coulter). Purified 216-bp amplicons were analyzed with an Agilent 2200 TapeStation D1000 (Agilent Technologies). Appropriate amplicons were prepared at 10 nM and loaded at 8 pM for sequencing on the Illumina MiSeq sequencer by using the Miseq Reagent Kit v2 300 cycles (Illumina).

Establishment of Barcoded Single-cell Clonal Lines. SAS-GL cells (4×10^5) were infected with the lentiviral barcode library. After 24 h, the medium was changed and selected with puromycin (1.5 μg/mL). After 3 days, single cells were sorted into 96-well

plates by using the SH800S cell sorter (Sony, Tokyo, Japan). Single-cell clonal lines were minimally expanded and frozen down. Barcode sequences for each clone were amplified with PCR by using KOD Plus (TOYOBO). The PCR products were purified with the QIAquick PCR Purification Kit (QIAGEN) and sequenced. The sequences of the primers used were as follows: forward primer: 5′-ATTTGGAATCACACGACCTGGATG-3′, reverse primer: 5′-GTACATGAAGCTGGTAGCCAGGATGT-3′, and sequencing primer: 5′-CACCATTATCGTTTCAGACCCACC-3′. We established 32 clones having unique barcode sequences.

Invasion Assay. Cells were plated on the top side of polycarbonate transwell filters coated with basement membrane extracts in the upper chamber of the CytoSelect 24-Well Cell Invasion Assay Kit (Cell Biolabs, San Diego, CA). Cells were suspended in medium without serum, and medium containing 10% serum was used as a chemoattractant in the lower chamber. After 24 h, cell fluorescence derived from the invading cells was read at 480/520 nm.

Colony Formation Assay. For the colony formation assay[3], cells (5×10^4 in total) cultured in 3D for 22 h were centrifuged and plated in standard 6-well tissue culture plates (Greiner Bio-One). Plates were kept in an incubator at 37 °C and 5% CO_2 for an additional 6 days without disturbance. Images of colonies derived from surviving cells were acquired by the IN Cell Analyzer 2200 (GE Healthcare, Pittsburgh, PA) and were analyzed by using the Developer Toolbox 1.9 software (GE Healthcare). Colonies with an area >25,000 μm^2 were counted. Also, for colonies with low growth capacity, we analyzed colonies with an area >200 μm^2.

Immunohistochemistry. Primary tumors from mice were fixed in 4% paraformaldehyde (PFA) and prepared according to a standard paraffin-embedding protocol. After deparaffinization in xylene and rehydration, antigens were retrieved by pressure cooking samples for 10 min in 1 mM EDTA buffer (pH 8.0). Slides were placed in 0.3% hydrogen peroxide/methanol for 30 min. After slides were rinsed with PBS for 5 min, they were incubated with blocking solution (1% bovine serum albumin [BSA]/TBS, 0.1% NaN_3) for 10 min. After sections were rinsed, they were incubated with anti-RFP antibody (1:200) (#600-401-379; Rockland Immunochemicals, Limerick, PA) diluted in PBS containing 1% BSA for 1.5 h at room temperature. After sections

were rinsed again, they were incubated with HRP-conjugated secondary antibody for 30 min. A color reaction was developed with HIGHDEF red IHC AP chromogen (#ADI-950-140-0030; Enzo Life Sciences, Burlington, ON, Canada) for 5 min. After sections were washed with deionized water, for detachment of 1st step antibodies, antigens were retrieved by microwaving for 3 min in 1 mM EDTA buffer (pH 8.0). The sections were incubated with blocking solution for 10 min and were then incubated with anti-TurboGFP antibody (1:1,000) (#PA5-22688; Thermo Fisher Scientific) diluted in PBS containing 1% BSA at room temperature for 1.5 h. After the sections were rinsed with PBS three times, they were incubated with HistoGreen (#E109; Linaris Biologische Produkte GmbH, Dossenheim, Germany) for 5 min.

For Ki-67 staining, tissue sections were autoclaved in 10 mmol/L citrate buffer (pH 6.0) for 20 min at 121 °C followed by cooling down for 20 min at room temperature. Slides were incubated with anti-Ki-67 antibody (1:100) (100130-MM22; Sino Biological, Beijing, China) diluted in PBS containing 1% BSA overnight at 4 °C. After slides were rinsed with PBS for 5 min, they were incubated for 1 h with HRP-conjugated secondary antibody. Chromogen was developed with 3,3-diaminobenzidine (Dako, Jena, Germany). All slides were lightly counterstained with hematoxylin for 10 s before dehydration and mounting. PBS and normal isotype-matched IgG were negative controls for primary antibodies.

RNA Interference Via shRNA. SAS cells with E-cadherin knockdown were generated via lentiviral infection with pLKO.1 puro containing shRNA sequences targeting E-cadherin (shEcad#1: AAGATAGGAGTTCTCTGATGC and shEcad#2: CGATTCAAAGTGGGCACAGAT). shEcad#1 was a gift of Bob Weinberg (Addgene plasmid #18801)[4]. Control cell lines were generated via lentiviral infection with pLKO.1-scramble shRNA, a gift of David Sabatini (Addgene plasmid #1864)[5]. Lentivirus was generated by transfection of HEK293T cells with pMD2.G and psPAX2. Transduced cells were selected by using puromycin (5 μg/mL). Knockdown efficiency was examined by using RT-qPCR and Western blot analyses.

Protein Extraction and Immunoblotting. Immunoblotting was performed as described previously[6]. Cells were washed once in ice-cold PBS and were then lysed by adding NP40 cell lysis buffer containing freshly added protease/phosphatase inhibitor cocktail (Sigma Aldrich). After incubation of the cell lysate on ice on a shaker for 15 min, the lysate was removed and centrifuged at 15,000 × *g* for 15 min to remove

insoluble material. Supernatants were stored at –70 °C until use. The protein concentration was determined by using a BCA kit (Pierce Chemical Co., Rockford, IL). Equal amounts of protein were fractionated via SDS-PAGE and transferred to PVDF membranes (Bio-Rad Laboratories, Hercules, CA). Membranes were blocked with 5% nonfat dry milk and 0.1% Tween 20 (Sigma Aldrich) in TBS (pH 7.4) and were then incubated overnight at 4 °C with antibodies against E-cadherin (#ab1416; Abcam, Cambridge, MA) or β-actin (#A5441; Sigma Aldrich) in 5% BSA (Sigma Aldrich) and 0.1% Tween 20 in TBS (pH 7.4). Several blots were cut prior to antibody hybridization for the simultaneous detection of different proteins in the same sample. After membranes were washed, they were incubated in HRP-conjugated secondary antibodies for 1 h. After this procedure, specific protein bands were detected via an enhanced chemiluminescence system (Amersham Pharmacia Biotech, Buckinghamshire, UK). Immunoblotting was repeated three times independently with similar results.

Immunofluorescence. Cells were fixed with 3.5% PFA for 10 min at room temperature and then cytospinned onto coated microscope slides (Matsunami Glass). Cells were permeabilized with 0.5% Triton X-100 (Sigma Aldrich) in PBS for 20 min and blocked with 1% BSA in PBS for 1 h. Slides were incubated with anti-myosin-IIA (1:100) (#909802; BioLegend, San Diego, CA), or anti-Ki-67 (1:100) antibodies diluted in PBS containing 1% BSA at 4 °C overnight, followed by incubation with Alexa Fluor 647 goat anti-rabbit IgG (1:100) (#A-21244; Thermo Fisher Scientific) at room temperature for 1 h. Slides were stained with DAPI (Sigma Aldrich) and mounted with ProLong Gold Antifade (#P36934; Thermo Fisher Scientific). Images were captured with the Olympus FV3000 confocal microscope (Olympus, Tokyo, Japan) using a 60× or 100× oil immersion objective. Myosin IIA distribution in cell aggregates generated in suspension culture and CTC clusters (n = 3 mice, ≥5 clusters/mouse) were evaluated. Z-series of focal planes through a contact area for each cell aggregate or clustered CTC were assessed.

Caspase Activation Assay. To measure caspase activity, cells plated in 96-well plates were treated with Bleb (20 μM) or DMSO for 3 h prior to analysis. Activities of caspase-3/7 were measured using a Caspase-Glo 3/7 assay kit (Promega) following the manufacturer's instructions.

mRNA-seq and Data Analysis. Total RNA was prepared from exponentially growing cells (Parental, clone H, and clone EE) by using Isogen (Nippon Gene, Toyama, Japan) according to the manufacturer's instructions. RNA integrity and concentration were assessed via the RNA Nano 6000 Assay Kit of the Bioanalyzer 2100 system (Agilent Technologies).

The library construction and sequencing were both performed by Annoroad Gene Technology Co., Ltd (Beijing, China). Briefly, sequencing libraries were prepared with the NEBNext Ultra II RNA Library Prep Kit for Illumina (NEB, Ipswich, MA) according to the manufacturer's instructions. The input material for RNA sample preparations was 2 μg of RNA per sample. Poly(A) mRNA was purified from total RNA by using poly-T oligo-attached magnetic beads, after which it was fragmented into short sequences. First-strand cDNA was synthesized by using random hexamer primer and RNase H. Second-strand cDNA synthesis was subsequently performed by using buffer, dNTPs, DNA polymerase I, and RNase H. The library fragments were purified with QiaQuick PCR kits (QIAGEN) and elution with EB buffer, and then terminal repair, A-tailing, and adapter added were carried out. The products of interest were retrieved and PCR was performed, and then the library was completed. cDNA libraries were sequenced on an Illumina Hiseq X (Illumina), and 150-bp paired-end reads were produced.

After sequencing was completed, we used FASTQC v0.11.8 software for an initial quality assessment and to detect common issues in RNA-seq data. Adaptor sequences and low-quality ends were removed with Trimmomatic (v0.38). Trimmed reads were aligned to a combined human (GRCh38) reference by using HISAT2 (v2.1.0). The expression level of each gene was quantified as fragments per kilobase of exons per million mapped reads (FPKM) by using both StringTie (v1.3.6) and Ballgown (v2.12.0) with default parameters. Genes whose expression was >1.5-fold higher in clone H but not higher in clone EE than in two other populations were defined as specifically up-regulated in clone H (1,852 genes). Similarly, up-regulated genes in clone EE (2,256 genes) were defined. GO enrichment analysis (Fisher's exact test with Bonferroni correction) was performed on the set of these genes via PANTHER 14.1[7,8]. Then, genes in identified GO terms ("molecular function" or "biological process") were categorized in terms of the GO biological process by using PANTHER. Lists and information about EBPs and their detailed functional categories were obtained from a previous report[9].

Analysis of the Gene Expression Dataset. We obtained a normalized expression dataset of breast cancer (GSE111065) from the NCBI Gene Expression Omnibus (GEO) public database to evaluate mRNA expression profiles in CTC clusters and single CTCs. GSEA—i.e., GSEA v4.0.1[10]—was used to identify the biological features that distinguished CTC clusters from single CTCs. Gene sets were compiled from MSigDB. Significant gene sets (FDR *q*-value < 0.18) for the C5 cellular component category were visualized as interaction networks with Cytoscape[11] and Enrichment Map software v3.2.1[12]. Black circles represented summarized gene set clusters based on AutoAnnotate[13]. The network map was manually obtained, with small annotated groups (≤4 and ≤7 gene sets for patient samples and xenograft samples, respectively) and nodes unconnected to any annotations being removed, which resulted in the simplified network map shown in Fig. 8a and Supplementary Fig. S8. Detailed GSEA results (FDR *q*-value < 0.25) for patient samples are given in the Supplementary Table S6. In the current analyses, no statistically significant enriched gene set existed for up-regulated genes in single CTCs, at least in categories such as cellular component (C5cc), molecular function (C5mf), and KEGG pathway (C2kegg).

The gene expression profiles and clinical information for patients with HNSCC were collected from TCGA via the cBioPortal (http://www.cbioportal.org)[14,15]. To validate the clinical significance of genes that were enriched in clone H (EBP-H; 54 genes), GSEA was performed for a subgroup of patients from the TCGA cohort with clinical information about recurrence (n = 374), pathological stage (n = 434), metastasis in lymph nodes or distant organs (pN or pM; n = 432), or histological grade (n = 483). Significant gene sets that were enriched were identified by using a nominal p value of <0.05 and FDR *q*-value of <0.25. We used 20 additional control lists of randomly selected gene sets, of comparable size (100 genes per each set) to address a possible caveat related to gene enrichment in tumors with various clinicopathological features. None of the random lists was significantly enriched in genes associated with a specific class of tumors (i.e., either LDR or NR) except for some gene sets for "Histological G3/4 vs. G1/2" tests, that still had a high FDR *q*-value (0.179-0.237).

Supplementary References

1. Zijlstra, A., Lewis, J., Degryse, B., Stuhlmann, H. & Quigley, J. P. The inhibition of tumor cell intravasation and subsequent metastasis via regulation of *in vivo* tumor cell motility by the tetraspanin CD151. *Cancer Cell* **13**, 221–234 (2008).
2. Bragado, P. et al. TGF-β2 dictates disseminated tumour cell fate in target organs through TGF-β-RIII and p38α/β signaling. *Nat. Cell Biol.* **15**, 1351–1361 (2013).
3. Schmidt, J. M. et al. Stem-cell-like properties and epithelial plasticity arise as stable traits after transient Twist1 activation. *Cell Rep.* **10**, 131–139 (2015).
4. Onder, T. T. et al. Loss of E-cadherin promotes metastasis via multiple downstream transcriptional pathways. *Cancer Res.* **68**, 3645–3654 (2008).
5. Sarbassov, D. D., Guertin, D. A., Ali, S. M. & Sabatini, D. M. Phosphorylation and regulation of Akt/PKB by the rictor-mTOR complex. *Science* **307**, 1098–1101 (2005).
6. Shinriki, S. et al. Loss of CYLD promotes cell invasion via ALK5 stabilization in oral squamous cell carcinoma. *J. Pathol.* **244**, 367–379 (2018).
7. Mi, H., Muruganujan, A., Ebert, D., Huang, X. & Thomas, P.D. PANTHER version 14: More genomes, a new PANTHER GO-slim and improvements in enrichment analysis tools. *Nucleic Acids Res.* **47**, D419–D426 (2019).
8. Ashburner, M. et al. Gene ontology: Tool for the unification of biology. *Nat Genet.* **25**, 25–29 (2000).
9. Guo, Z. et al. E-cadherin interactome complexity and robustness resolved by quantitative proteomics. *Sci. Signal.* **7**, rs7 (2014).
10. Subramanian, A. et al. Gene set enrichment analysis: A knowledge-based approach for interpreting genome-wide expression profiles. *Proc. Natl. Acad. Sci. USA* **102**, 15545–15550 (2005).
11. Shannon, P. et al. Cytoscape: A software environment for integrated models of biomolecular interaction networks. *Genome Res.* **13**, 2498–2504 (2003).
12. Merico, D., Isserlin, R., Stueker, O., Emili, A. & Bader, G. D. Enrichment map: A network-based method for gene-set enrichment visualization and interpretation. *PLoS One* **5**, e13984 (2010).
13. Kucera, M., Isserlin, R., Arkhangorodsky, A. & Bader, G. D. AutoAnnotate: A Cytoscape app for summarizing networks with semantic annotations. *F1000Res.* **5**, 1717 (2016).
14. Cerami, E. et al. The cBio cancer genomics portal: An open platform for exploring multidimensional cancer genomics data. *Cancer Discov.* **2**, 401–404, (2012).

15. Gao, J. et al. Integrative analysis of complex cancer genomics and clinical profiles using the cBioPortal. *Sci. Signal.* **6**, pl1 (2013).
